# Inflammation Does Not Mediate Relationships between BMI/BMI-for-Age *Z*-Score and Retinol or Retinol-Binding Protein in Adult Women or Young Children without Underweight, Wasting, or Malaria: An Analysis of 24 Surveys

**DOI:** 10.1016/j.cdnut.2026.107684

**Published:** 2026-04-02

**Authors:** Jennie N Davis, Anne Williams, Charles D Arnold, Yaw Addo, Brietta M Oaks, James P Wirth, Parminder S Suchdev, Melissa F Young, Reina Engle-Stone

**Affiliations:** 1 Institute for Global Nutrition, University of California, Davis, Davis, CA, United States; 2 Department of Global Health, Rollins School of Public Health, Emory University, Atlanta, GA, United States; 3 Department of Nutrition, University of California, Davis, Davis, CA, United States; 4 Division of Nutrition, Physical Activity and Obesity, Centers for Disease Control and Prevention, Chamblee, GA, United States; 5 Department of Nutrition, University of Rhode Island, Kingston, RI, United States; 6 GroundWork, 7306 Fläsch, Switzerland; 7 Department of Pediatrics and Global Health, Emory University, Atlanta, GA, United States; 8 Division of Global Health Protection, Centers for Disease Control and Prevention, Atlanta, GA, United States

**Keywords:** vitamin A, inflammation, nutrition assessment, overweight, obesity, mediation analysis, BRINDA

## Abstract

**Background:**

The extent to which inflammation from overweight or obesity influences interpretation of commonly used vitamin A status biomarkers is uncertain.

**Objectives:**

We examined relationships among weight status, inflammation, and retinol or retinol-binding protein (RBP) among women (15–49 y) and children (6–59 mo) with normal weight to overweight or obesity.

**Methods:**

Cross-sectional data were separately analyzed from 24 surveys representing 24 countries (*n* = 16,771 women in 13 surveys and *n* = 24,707 children in 22 surveys) from the Biomarkers Reflecting Inflammation and Nutritional Determinants of Anemia (BRINDA) project, excluding observations with underweight, wasting, pregnancy, or malaria. Relationships were assessed between BMI (women) or BMI-for-age *z*-score (BAZ, children), inflammatory biomarkers C-reactive protein (CRP) and/or α1-acid glycoprotein (AGP), and retinol or RBP by linear regression. We also examined potential mediation by CRP and/or AGP in relationships between BMI/BAZ and retinol/RBP with structural equation modeling. Regression and mediation models accounted for complex survey designs and were adjusted for potential confounders.

**Results:**

Among women, BMI was positively associated with retinol/RBP in 5 of 13 surveys and positively associated with CRP and/or AGP in 10 of 13 surveys; associations between biomarkers of inflammation and retinol/RBP varied in direction and magnitude. Among children, BAZ was positively associated with retinol/RBP in 3 of 22 surveys and positively associated with CRP in 1 survey (none with AGP); inflammation biomarkers were consistently negatively associated with retinol/RBP. In 3 of 13 women’s surveys and 1 of 22 children’s surveys, inflammation partially mediated the relationship between BMI/BAZ and retinol/RBP; however, the direction of associations varied.

**Conclusions:**

In these surveys limited to individuals without undernutrition or malaria, inflammation associated with overweight or obesity does not appear to impact vitamin A assessment when measured with retinol/RBP. However, findings support measurement of biomarkers reflecting inflammation (of any source) to interpret vitamin A status, particularly among children.

## Introduction

Vitamin A (VA) deficiency affects ∼30% of children aged <5 y [1] and 15% of pregnant women globally [2], making addressing this deficiency an ongoing public health priority [3]. The primary cause of VA deficiency globally is insufficient diet, that is, chronically low intakes of VA-containing foods that result in the body’s inability to meet its physiological VA needs, including for normal cellular and metabolic functions, tissue growth, and infection resistance [3]. Historically, VA deficiency has been more widespread in low- and middle-income countries where diets low in bioavailable VA are common, and deficiency may exacerbate the severity of infectious illnesses common to these areas, such as diarrhea and measles [[2], [3], [4]].

Concentrations of serum or plasma retinol and retinol-binding protein (RBP) are frequently measured biomarkers of VA status in epidemiological studies, with RBP often used as a proxy for retinol [5,6]. RBP is primarily synthesized in the liver, the principal storage site of VA, which homeostatically regulates retinol and RBP secretion; in circulation, RBP serves as the primary retinol transporter [3]. In addition to hepatic production, RBP is also secreted by adipose tissue [7,8] through mechanisms that may be independent of VA metabolism [9]. Circulating RBP concentrations can increase with excess adiposity [7,10], potentially complicating VA assessment in individuals with obesity [9,11].

As a negative acute-phase protein, plasma RBP concentrations decrease during inflammatory conditions such as illness, infection, or trauma due to reduced hepatic secretion and increased renal losses, leading to lower circulating concentrations of both retinol and RBP [3]. Obesity is also an inflammatory state, characterized by chronic, low-grade inflammation [12,13]. Several proinflammatory cytokines linked to adipose tissue, including IL-6, TNF-α, C-reactive protein (CRP), and many others, are inflammatory mediators that may stimulate immune cells and trigger an inflammatory response [[14], [15], [16]]. Inflammation associated with overweight or obesity may influence circulating retinol and RBP concentrations through overlapping but distinct pathways from those of infection-related inflammation [8,9]. Although most literature on VA biomarkers and inflammation in children has focused on infectious illnesses, a growing body of research on VA biomarkers and chronic disease has reported associations between retinol or RBP and cardiovascular disease, obesity, and insulin resistance [8,11,[17], [18], [19]]. Together, these findings highlight the need to understand whether obesity-related inflammation contributes to reductions in circulating retinol and RBP, as observed during the acute-phase response, and how this may affect interpretation of VA status in populations experiencing high or rising obesity prevalence.

The negative association between inflammation and retinol or RBP has been documented in preschool children and adult women [6]. The Biomarkers Reflecting Inflammation and Nutritional Determinants of Anemia (BRINDA) project previously established that adjusting for inflammation with a regression correction among children consistently decreased the estimated prevalence of VA deficiency (median: −8.2%; range: −16.0% to −3.4%) [6]; however, the association between inflammation and VA biomarkers among adult women was less consistent, and adjusting for inflammation was not recommended [5,6,20].

Previous BRINDA project research also examined the prevalence and independence of intraindividual overweight or obesity and micronutrient deficiencies among children and adult women [21,22] and reported that overweight or obesity was generally not associated with VA deficiency for either group. However, these analyses did not explore weight status as a source of inflammation, which could potentially influence VA status assessment, and to our knowledge, no studies have assessed whether the inflammation associated with overweight or obesity may impact population VA assessment. Therefore, using data from 24 surveys from the BRINDA project, we aimed to determine the relationships between weight status [BMI (in kg/m^2^) or BMI-for-age *z*-score (BAZ)], inflammation biomarkers of CRP and/or α1-acid glycoprotein (AGP), and VA biomarkers (retinol or RBP) among adult women and young children with normal weight to overweight or obesity, and to examine whether inflammation mediates the relationship between weight status and VA biomarkers.

## Methods

### Data source and inclusion criteria

The BRINDA project includes a harmonized data set of nationally or regionally representative surveys. Previous publications document the BRINDA project methods, including criteria for survey and variable inclusion, anthropometric calculations, and biochemical collection methodology [23,24]. For these secondary analyses, we analyzed 13 data sets from 13 countries with deidentified data on nonpregnant women of reproductive age 15–49 y (women) and 22 data sets from 20 countries with deidentified data on preschool-aged children 6–59 mo (children; 24 total surveys from 24 countries). Our inclusion and exclusion criteria correspond to previous BRINDA project analyses [21,22,25], with the exception that for our analyses the surveys must have measured serum or plasma retinol or RBP and included a marker of inflammation (CRP and/or AGP). Observations were excluded with the following: BMI <18.5 (women); BAZ or weight-for-height *z*-score <−2 SD (children); height or weight outside the ranges of 101.6–219.9 cm and 22.7–222.2 kg, respectively (women) [22]; BAZ <−5 SD or >5 SD (children) [21]; zero values for retinol or RBP; self-reported pregnancy (women; *n* = 5 surveys did not include a pregnancy variable and it is possible data from pregnant women were not excluded from these) (Supplemental Table 1); or a positive malaria result [*n* = 5 (women) and *n* = 9 (children) surveys measured malaria]. As in prior analyses [25], we excluded observations with underweight, wasting, or positive malaria test result to minimize the influence of inflammation due to infectious disease; that is, we assume that inflammation among those who are underweight or wasted is more likely to be due to infections or causes other than adiposity.

After application of exclusion criteria, the analytical sample in 10 of 13 women’s surveys and 18 of 22 children’s surveys included ≥40% of the original observations. As in previous analyses [25], we did not apply a sample size cutoff for excluding surveys, but did ensure that all surveys met the minimum analytical sample size required for mediation analysis: a minimum of 10 observations per linear relation [26]. In this data set, the smallest survey sample size was Burkina Faso for both women (*n* = 61) and children (*n* = 60). Both surveys included CRP and AGP as inflammatory markers, meaning there were 5 linear relations for mediation analyses, necessitating a minimum sample size of 50. Supplemental Tables 1 and 2 report the proportion of observations excluded overall and by individual exclusion criterion in each survey.

### Variable definitions

The outcome variables were retinol (in micromoles per liter) or RBP (micromoles per liter), CRP (milligrams per liter), and AGP (grams per liter); the predictor variables were BMI (women) or BAZ (children), CRP (milligrams per liter), and AGP (grams per liter). Among women, 10 surveys measured retinol and 8 measured RBP, including 4 surveys that measured both biomarkers. Among children, retinol and RBP were each measured in 13 surveys, including 4 surveys that measured both. For surveys that measured both retinol and RBP, we selected the biomarker with the greater number of observations prior to application of our exclusion criteria. CRP was measured in all women’s and 19 children’s surveys, AGP in 10 women’s and 19 children’s surveys, and both CRP and AGP were measured in 10 women’s and 16 children’s surveys. For the mediation analysis, the outcome variable was either retinol or RBP, the predictor variable was BMI or BAZ, and the mediating variables were CRP and/or AGP (both were included if the survey included both variables).

Covariates available and selected for analysis are listed by survey in Supplemental Table 3. Covariates were defined as reported by survey representatives and were selected for analysis based on expected relationships with VA, as well as potential confounders of the relationship between BMI, inflammation, and VA. Covariates included age [years (women) or months (children)]; sex (children only); urban or rural residence; socioeconomic status (SES, an ordinal 3-category variable of low, medium, and high SES categories from the harmonized BRINDA project data set) [23]; water and sanitation (defined by the WHO/UNICEF Joint Monitoring Program as access to an improved or unimproved water source or toilet facility) [27,28]; highest level of education level completed by the respondent (women) or mother (children) except in surveys that reported household head education level [Burkina Faso and Colombia (women and children)]; and whether or not VA supplementation was received in the last 6 mo (children only, 9 surveys).

Categorical variables were defined as follows: BMI (women) categorized as normal (18.5–24.9) and overweight or obesity (≥25.0) [29]; BAZ (children) categorized as normal (−2 to 2 SD) and overweight or obesity (>2 SD) [30]; and any inflammation defined as CRP >5 mg/L and/or AGP >1 g/L. For both women and children, VA deficiency was defined as retinol or RBP of <0.7 μmol/L [31], and the ratio of retinol to RBP was assumed to be 1:1, consistent with prior BRINDA project publications [5,6] and recognizing that, in practice, this ratio may vary. Among women only, VA insufficiency (<1.05 μmol/L) [32] is also presented due to the small number of observations below the deficiency cutoff [6]. After applying our exclusion criteria, ≤3% of women in 10 of 13 surveys were VA deficient.

As the etiology of inflammation may differ by context, with greater risk of infectious illness in lower-income countries and greater prevalence of overweight and obesity among higher-income contexts [33,34], we present results grouped by World Bank country income–level classifications (CICs) based on their classification at the time the survey was conducted [35]. The country income groups are presented for qualitative interpretation only (i.e., no formal statistical testing was completed between country groupings).

### Statistical analyses

Data from each survey were analyzed separately and by group (women or children), accounting for complex survey designs (cluster and strata) when necessary and using study survey weights. *P* values were considered statistically significant at *P* < 0.050, and all analyses were conducted in SAS version 9.4 (SAS Institute) or STATA version 16 (StataCorp).

For continuous variables, means (95% CI) are presented, with geometric means presented for CRP, AGP, retinol, and RBP due to skewed distributions. Age is presented as the median (IQR). Categorical variables are presented as percent (95% CI). Among children only for presentation of descriptive results (prevalence estimates), retinol and RBP concentration and prevalence of VA deficiency were adjusted for inflammation following the BRINDA project regression correction approach [23].

#### Bivariate and multivariable analyses

Following methodology of previous analyses of these data [25], we generated and applied a random value between 0 and the lowest value for CRP for surveys in which >30% of the analytical sample's CRP values had a single low value [Afghanistan, Colombia, and Zambia (children)]. For example, 65% (*n* = 2418) of the CRP values in the survey from Colombia were reported as 0.2; for these observations, we generated and applied a random value between 0 and 0.2 [36,37]. Retinol, RBP, CRP, and AGP were natural log (*ln*) transformed to achieve normal distributions and the residuals visually examined for all surveys. Sensitivity analyses compared the Spearman rank correlation coefficient with the bivariate linear regression estimate for any surveys with abnormal residual distributions after transformation, with results examined to determine whether linear regression analyses should proceed. For this study, regression analyses in children’s surveys were conducted with the unadjusted retinol or RBP variable.

In each survey, we assessed the following bivariate linear regression models:

1. *Y*(*ln*Retinol or RBP) = *B*_0_ + *B*_1_(BMI)∗
2. *Y*(*ln*CRP) = *B*_0_ + *B*_1_(BMI)∗
3. *Y*(*ln*AGP) = *B*_0_ + *B*_1_(BMI)∗
4. *Y*(*ln*Retinol or RBP) = *B*_0_ + *B*_1_(*ln*CRP)
5. *Y*(*ln*Retinol or RBP) = *B*_0_ + *B*_1_(*ln*AGP)
∗ Children’s models used BAZ in place of BMI.

In separate bivariate linear regression analyses (unadjusted models), each covariate was tested with each outcome variable; marginally significant covariates (*P* < 0.100) were then included in a multivariable adjusted model. Models were assessed for collinearity with variance inflation factors (>5) and tolerance (>0.1). To evaluate individual associations between CRP or AGP and retinol or RBP, inflammation variables were analyzed in separate models, not combined into a single variable. Regression results are presented as percent change in the dependent variable for every 1-unit change in the independent variable, with their specific units of measurement (retinol or RBP: μmol/L; CRP: mg/L; AGP: g/L). In the case of equations 4 and 5, the independent biomarker was also log transformed and thus also represents relative change.

#### Mediation analysis

To test the hypothesis that inflammation mediates part or all of the relationship between BMI or BAZ and retinol or RBP among women or children with normal to elevated BMI/BAZ, we conducted mediation analyses with structural equation modeling procedures in STATA following a path diagram (Supplemental Figure 1, the path through CRP or AGP was not included for surveys without the corresponding variable). Given the cross-sectional study design, models can be interpreted as statistical decompositions of observed associations rather than tests of causal or biological mediation; however, we retain the term “effect” for simplicity in discussing mediation results. Mediation analysis interpretation was: total effect = the effect of BMI/BAZ on VA biomarkers (retinol or RBP) not controlling for inflammation biomarkers (CRP and/or AGP); direct effect = the effect of BMI/BAZ on VA biomarkers controlling for inflammation biomarkers; and indirect effect = the effect of BMI/BAZ on VA biomarkers mediated by the effect of CRP and/or AGP. Mediation was considered present if both the total effect and the indirect effect were significant at *P* < 0.050 [38,39]. Mediation analyses were adjusted for complex survey designs through application of survey weights. Mediation estimates were exponentiated, and results are presented as percent change in retinol or RBP concentration for every 1-unit change in BMI or BAZ. Each structural equation model was adjusted for the same covariates used in the corresponding survey’s bivariate models. Mediation analyses in children’s surveys were conducted with the unadjusted retinol or RBP variable.

#### Sensitivity analyses

To further examine the potential contribution of sources of inflammation to the relationship between VA biomarkers and BMI or BAZ, we conducted 3 sensitivity mediation analyses. In data sets with available malaria data (*n* = 4 women’s surveys and *n* = 8 children’s surveys; Burkina Faso surveys were not included as all malaria-positive observations were excluded with our exclusion criteria for underweight/wasting), we evaluated the relationship among BMI or BAZ, VA biomarkers, and inflammation among all individuals compared with those without a positive malaria result. Observations with morbidity symptoms (i.e., reported fever or diarrhea) were not excluded from the analytic sample because the variables were not reported uniformly across surveys, and prior BRINDA project analyses showed that reported morbidity was not consistently associated with inflammation [40]. However, we conducted another sensitivity mediation analysis in a subset of surveys that excluded these observations [fever or diarrhea in the past 24 h (Kenya 2010, children) or past 2 wk (Liberia, children; Côte d’Ivoire and Malawi, women)]; results were qualitatively compared. A third sensitivity mediation analysis was conducted among 4 surveys that measured both retinol and RBP in both women and children (Burkina Faso, Cameroon, Malawi, and Nigeria) to examine potential differences between VA biomarkers and their relationship with BMI or BAZ and inflammatory biomarkers.

### Ethics approval

All BRINDA project data sets were deidentified and had appropriate ethical approvals at the time of data collection. As such, this analysis was considered nonhuman subjects research and did not require review by an institutional review board, consistent with applicable federal law and Centers for Disease Control and Prevention policy.

## Results

### Participant characteristics and nutrition and health status

Among women, after application of our exclusion criteria, the analytical sample size ranged from 61 (Burkina Faso) to 4946 (Pakistan), with a total sample size of 16,771 (Supplemental Table 4). Across surveys, the median age was ∼30 y. The proportion of the analytical sample residing in urban areas (assessed in 9/13 surveys) ranged from 10.3% (Malawi) to 62.8% (Cameroon); the survey from Nigeria contained observations from rural areas only (Supplemental Table 4). In surveys with SES data (assessed in 11/13 surveys) and after applying the exclusion criteria, >60% of the population was classified as high SES in each survey (Supplemental Table 4).

Among women, the mean prevalence of overweight and obesity in surveys from upper-middle–income and high-income countries was more than double the mean prevalence of overweight and obesity in surveys from low-income and low-middle–income countries (54.2% and 25.7%, respectively), with the survey from Azerbaijan reporting the greatest prevalence of overweight and obesity (57.3%; 95% CI: 54.7, 59.8) (Table 1). The prevalence of VA insufficiency was <8% in upper-middle–income and high-income countries and ranged from 5.8% (Vietnam) to 63.7% (Pakistan) in low- and low-middle–income countries. In this sample, excluding individuals with underweight or malaria, the mean prevalence of inflammation (elevated CRP and/or AGP) was similar among surveys from combined upper-middle–income and high-income countries (25.6%) and low- and low-middle–income countries (27.8%) (Table 1).

**TABLE 1:** Biological and nutritional characteristics for women (15–49 y) with normal weight to overweight/obesity by survey: BRINDA project^,^^1^.

CIC^2^	Country, survey year	*n*	BMI, mean (95% CI)	Overweight/obesity^3^ (%)	CRP (mg/L)	AGP (g/L)	Any inflammation^4^ (%)	RBP or SR^5^ (μmol/L)	VA deficiency^6^ (%)	VA Insufficiency^6^ (%)
Low	Afghanistan, 2013	571	25.1 (24.6, 25.6)	42.1 (36.0, 48.1)	0.7 (0.5, 0.8)	0.7 (0.7, 0.7)	18.3 (14.0, 22.5)	1.1 (1.0, 1.2)	11.0 (7.5, 14.5)	42.2 (35.0, 49.3)
	Burkina Faso, 2010	61	20.8 (20.0, 21.7)	3.1 (0.0, 7.8)	1.8 (1.3, 2.2)	1.2 (1.1, 1.3)	74.5 (63.5, 85.5)	0.9 (0.8, 1.1)	18.7 (9.8, 27.6)	63.4 (41.9, 85.0)
	Cambodia, 2014	609	22.9 (22.5, 23.3)	22.5 (17.1, 27.9)	0.8 (0.7, 0.9)	0.7 (0.7, 0.8)	35.9 (25.9, 45.8)	2.0 (1.8, 2.1)	3.0 (1.6, 4.3)	9.2 (6.6, 11.8)
	Côte D’Ivoire, 2007	706	23.5 (23.1, 23.8)	26.0 (22.3, 29.8)	1.5 (1.3, 1.7)	0.8 (0.8, 0.8)	31.9 (27.9, 35.9)	1.5 (1.4, 1.5)	0.7 (0.1, 1.3)	13.2 (10.2, 16.1)
	Malawi, 2016	595	22.8 (22.2, 23.4)	17.7 (13.3, 22.0)	0.7 (0.6, 0.8)	0.6 (0.6, 0.6)	11.7 (8.4, 15.1)	1.4 (1.3, 1.4)	2.7 (0.7, 4.7)	14.2 (10.5, 17.8)
	Papua New Guinea, 2005	692	23.3 (22.9, 23.7)	24.4 (19.6, 29.2)	0.4 (0.3, 0.5)	1.6 (1.5, 1.6)	23.6 (20.0, 27.3)	1.6 (1.5, 1.6)	0.4 (0.0, 1.0)	6.7 (4.5, 8.8)
Low-middle	Cameroon, 2009	594	24.7 (24.3, 25.1)	39.0 (34.4, 43.6)	0.9 (0.8, 1.1)	0.7 (0.7, 0.8)	16.3 (13.2, 19.4)	1.4 (1.3, 1.4)	1.4 (0.5, 2.3)	12.8 (9.7, 15.9)
	Nigeria, 2012	506	24.2 (23.6, 24.7)	35.2 (29.5, 40.9)	1.6 (1.4, 1.8)	0.7 (0.7, 0.8)	25.7 (21.6, 29.8)	1.6 (1.6, 1.7)	1.0 (0.8, 2.0)	8.7 (5.6, 11.8)
	Pakistan, 2011	4946	24.4 (24.2, 24.5)	36.5 (34.3, 38.3)	1.0 (0.9, 1.0)	0.8 (0.8, 0.8)	32.6 (36.0, 40.8)	0.8 (0.7, 0.8)	38.4 (36.0, 40.8)	63.7 (61.3, 66.2)
	Vietnam, 2010	1138	21.7 (21.6, 21.9)	10.2 (8.3, 12.0)	0.9 (0.8, 0.9)	—	7.1 (5.9, 8.4)	1.7 (1.6, 1.7)	1.1 (0.6, 1.7)	5.8 (4.4, 7.2)
Upper-middle	Azerbaijan, 2013	2528	26.8 (26.5, 27.1)	57.3 (54.7, 59.8)	1.1 (1.1, 1.2)	0.9 (0.8, 0.9)	34.9 (32.5, 37.4)	1.5 (1.5, 1.5)	0.4 (0.1, 0.7)	7.8 (6.5, 9.2)
High	United Kingdom, 2014	836	26.4 (25.8, 27.0)	49.0 (43.9, 54.2)	2.2 (2.0, 2.4)	—	15.5 (12.2, 18.9)	1.6 (1.5, 1.7)	1.1 (0.0, 2.2)	7.3 (4.1, 10.5)
	United States, 2006	2989	27.9 (27.4, 28.5)	56.3 (52.7, 59.9)	1.8 (1.7, 1.9)	—	26.3 (24.1, 28.5)	1.8 (1.7, 1.8)	0.3 (0.1, 0.6)	3.0 (2.2, 3.7)

Abbreviations: AGP, α1-acid glycoprotein; BRINDA, Biomarkers Reflecting Inflammation and Nutritional Determinants of Anemia; CIC, country income classification; CRP, C-reactive protein; RBP, retinol-binding protein; SR, serum retinol; VA, vitamin A.

1 CRP, AGP, RBP, and SR values are presented as geometric mean (95% CI) due to nonnormal distributions. All estimates account for survey design variables (cluster, strata, and weight); — indicates the variable was unavailable in that survey. Inclusion criteria were BMI ≥18.5, not pregnant, and a negative malaria test result to reduce potential confounding from inflammation due to illness or infection.

2 CIC was defined according to the World Bank definition for the year in which the survey was conducted [35].

3 Overweight/obesity defined as a BMI ≥25.0.

4 Any inflammation defined as CRP >5 mg/L or AGP >1 g/L.

5 RBP or SR measured in plasma or serum, as reported in the survey. Surveys that measured RBP were as follows: Cambodia, Côte D’Ivoire, Malawi, Papua New Guinea, Cameroon, Nigeria, and Azerbaijan. Surveys that measured SR were as follows: Afghanistan, Burkina Faso, Pakistan, Vietnam, United Kingdom, and United States.

6 VA deficiency defined as serum or plasma RBP or SR concentration of <0.7 μmol/L. VA insufficiency defined as serum or plasma RBP or SR of <1.05 μmol/L [31,32].

Among children, after application of our exclusion criteria, the analytical sample size ranged from *n* = 60 (Burkina Faso) to *n* = 5896 (Pakistan), with a total sample size of 24,707 (Supplemental Table 5). Most surveys (*n* = 12 of 22) included children 6–59 mo of age (Supplemental Table 5). The proportion residing in urban areas (assessed in 19/22 surveys) ranged from 9.1% (Philippines) to 72.2% (Mexico); the surveys from Kenya (2007, 2010) and Nigeria contained observations from rural areas only (Supplemental Table 5). Among surveys that measured SES (assessed in 16/22 surveys), the proportion of the analytical sample classified as high SES ranged from 16.0% (Philippines) to 95.1% (Afghanistan) (Supplemental Table 5).

Among children, prevalence of overweight and obesity was <10% in all surveys, except in Mongolia (10.5%), Nigeria (11.7%), and Azerbaijan (14.5%) (Table 2). The prevalence of inflammation-adjusted VA deficiency varied across surveys, ranging from <1% in Nicaragua and the Philippines to 42.8% in Zambia and 51.0% in Pakistan (Table 2). The mean prevalence of any inflammation across all upper-middle–income surveys was 20.5% and 43.6%, across all surveys from low- and low-middle–income countries (Table 2).

**TABLE 2:** Biological and nutritional characteristics for children (6–59 mo) with normal weight to overweight/obesity by survey: BRINDA project^1^.

CIC^2^	Country, survey year	*n*	BAZ	Overweight/obesity^3^ (%)	CRP (mg/L)	AGP (g/L)	Any inflammation^4^ (%)	RBP or SR (μmol/L)^5^	VA deficiency^6^ (%)	VAS^7^ (%)
Low	Afghanistan, 2013	586	0.2 (0.1, 0.3)	7.0 (3.7, 10.3)	0.3 (0.2, 0.3)	0.8 (0.8, 0.8)	26.5 (21.5, 31.6)	0.7 (0.7, 0.8)	37.8 (32.5, 43.0)	43.3 (35.5, 51.2)
	Bangladesh, 2010	1179	−0.6 (−0.7, −0.5)	2.1 (1.3, 2.9)	0.8 (0.6, 0.9)	0.9 (0.9, 0.9)	34.4 (30.8, 38.1)	1.0 (1.0, 1.0)	5.3 (3.4, 7.2)	—
	Bangladesh, 2012	360	−0.4 (−0.6, −0.3)	3.8 (0.7, 6.9)	0.7 (0.6, 0.9)	0.8 (0.8, 0.9)	27.6 (19.9, 35.4)	1.0 (0.9, 1.0)	5.6 (2.2, 8.9)	78.3 (69.5, 87.1)
	Burkina Faso, 2010	60	−0.1 (−0.5, 0.3)	1.6 (0, 5.4)	5.2 (3.0, 7.4)	1.6 (1.5, 1.7)	91.9 (86.0, 97.8)	1.0 (0.9, 1.2)	13.4 (4.4, 22.5)	—
	Cambodia, 2014	599	−0.4 (−0.5, −0.4)	0.8 (0, 1.7)	0.6 (0.5, 0.7)	0.8 (0.7, 0.9)	38.5 (30.5, 46.5)	1.4 (1.3, 1.4)	6.3 (4.1, 8.5)	—
	Côte d’Ivoire, 2007	435	0.1 (0.0, 0.2)	6.2 (3.7, 8.6)	2.0 (1.6, 2.4)	1.1 (1.1, 1.1)	59.3 (54.3, 64.3)	1.3 (1.2, 1.3)	2.0 (0.7, 3.4)	—
	Kenya, 2007	665	0.2 (0.2, 0.3)	4.2 (2.5, 5.9)	1.0 (0.8, 1.2)	1.1 (1.1, 1.1)	59.4 (54.6, 64.2)	1.1 (1.0, 1.1)	5.6 (3.9, 7.2)	—
	Kenya, 2010	551	0.3 (0.2, 0.3)	4.5 (3.0, 6.1)	0.9 (0.7, 1.1)	1.0 (0.9, 1.0)	47.9 (42.0, 53.8)	1.1 (1.0, 1.1)	7.8 (5.7, 10.0)	—
	Liberia, 2011	956	−0.1 (−0.2, 0.01)	2.5 (1.4, 3.7)	1.4 (1.2, 1.6)	1.0 (0.9, 1.0)	50.4 (45.9, 54.8)	1.1 (1.0, 1.1)	5.5 (4.0, 7.0)	90.9 (88.4, 93.4)
	Malawi, 2016	748	0.1 (−0.1, 0.2)	5.3 (2.9, 7.8)	1.0 (0.8, 1.2)	1.0 (1.0, 1.1)	49.0 (42.5, 55.6)	1.0 (1.0, 1.1)	8.0 (5.1, 10.8)	70.1 (62.9, 77.2)
	Mongolia, 2006	202	0.8 (0.7, 0.9)	10.5 (6.9, 15.1)	—	0.8 (0.8, 0.9)	24.7 (19.4, 30.7)	0.8 (0.7, 0.8)	26.2 (20.2, 32.3)	—
	Nicaragua, 2005	1403	0.5 (0.4, 0.7)	6.2 (4.2, 8.2)	—	0.8 (0.8, 0.8)	24.1 (20.6, 27.6)	1.3 (1.3, 1.3)	0.7 (0.3, 1.1)	68.6 (64.2, 73.0)
	Papua New Guinea, 2005	802	0.2 (0.1, 0.3)	5.1 (2.7, 7.5)	1.3 (1.1, 1.6)	1.0 (1.0, 1.1)	56.3 (51.9, 60.8)	1.0 (1.0, 1.0)	10.1 (7.8, 12.5)	33.3 (23.4, 43.2)
	Zambia, 2009	313	0.4 (0.3, 0.6)	5.4 (2.4, 8.5)	1.6 (0.9, 2.2)	1.0 (0.9, 1.0)	71.6 (67.4, 75.7)	0.7 (0.7, 0.8)	42.8 (34.7, 51.0)	—
Low-middle	Cameroon, 2009	556	0.4 (0.3, 0.5)	4.2 (2.0, 6.3)	1.3 (1.1, 1.5)	0.9 (0.9, 0.9)	37.7 (32.7, 42.7)	1.0 (1.0, 1.0)	11.1 (8.3, 14.0)	27.0 (22.1, 32.0)
	Nigeria, 2012	283	0.4 (0.2, 0.7)	11.7 (6.1, 17.2)	2.0 (1.6, 2.5)	1.0 (0.9, 1.0)	56.2 (48.8, 63.6)	1.0 (1.0, 1.1)	11.7 (7.8, 12.5)	—
	Pakistan, 2011	5896	−0.1 (−0.1, −0.02)	5.4 (4.7, 6.1)	—	0.9 (0.9, 0.9)	35.3 (33.7, 36.9)	0.6 (0.6, 0.6)	51.0 (48.6, 53.3)	—
	Philippines, 2011	1656	−0.1 (−0.2, −0.01)	2.1 (1.0, 3.3)	0.7 (0.6, 0.9)	0.8 (0.8, 0.8)	25.9 (22.3, 29.4)	1.2 (1.2, 1.2)	0.8 (0.3, 1.3)	62.8 (58.8, 66.9)
	Vietnam, 2010	329	−0.1 (−0.3, −0.02)	4.3 (2.0, 6.5)	0.7 (0.6, 0.8)	—	12.5 (9.7, 15.3)	1.2 (1.2, 1.3)	5.5 (2.9, 8.0)	—
Upper-middle	Azerbaijan, 2013	987	0.9 (0.8, 1.0)	14.5 (11.7, 17.3)	0.3 (0.3, 0.4)	0.8 (0.8, 0.9)	30.8 (27.0, 34.6)	1.1 (1.0, 1.1)	6.2 (3.5, 8.8)	2.1 (1.0, 3.3)
	Colombia, 2010	3711	0.4 (0.3, 0.4)	3.7 (2.9, 4.4)	0.3 (0.3, 0.4)	—	18.9 (17.2, 20.7)	0.9 (0.9, 0.9)	19.8 (18.0, 21.7)	—
	Mexico, 2012	2430	0.6 (0.5, 0.6)	8.5 (6.7, 10.3)	0.5 (0.4, 0.6)	—	11.8 (9.3, 14.2)	1.0 (1.0, 1.0)	7.0 (5.4, 8.6)	—

Abbreviations: AGP, α1-acid glycoprotein; BAZ, BMI-for-age *z*-score; BRINDA, Biomarkers Reflecting Inflammation and Nutritional Determinants of Anemia; CIC, country income classification; CRP, C-reactive protein; RBP, retinol-binding protein; SR, serum or plasma retinol; VAS, vitamin A supplementation; WHZ, weight-for-height *z*-score.

1 CRP, AGP, SR, and RBP values are presented as geometric mean (95% CI) due to nonnormal distributions. All estimates account for survey design variables (cluster, strata, and weight), except Mongolia, which followed a simple random sampling design; — indicates the variable was unavailable in that survey. Inclusion criteria were as follows: BAZ or WHZ ≥−2 SD and a negative malaria test result to reduce potential confounding from inflammation due to illness or infection.

2 CIC was defined according to the World Bank definition for the year in which the survey was conducted [35].

3 Overweight/obesity defined as a BAZ of >2 SD.

4 Any inflammation defined as CRP of >5 mg/L or AGP >1 g/L.

5 RBP or SR measured in plasma or serum, as reported in the survey. Surveys that measured RBP were as follows: Bangladesh 2010, Cambodia, Côte d’Ivoire, Kenya 2007 and 2010, Liberia, Malawi, Papua New Guinea, Cameroon, Philippines, and Azerbaijan. Surveys that measured SR were as follows: Afghanistan, Bangladesh 2012, Burkina Faso, Mongolia, Nicaragua, Zambia, Nigeria, Pakistan, Vietnam, Colombia, and Mexico.

6 VA deficiency defined as RBP or SR concentration of <0.7 μmol/L [31], adjusted for inflammation using the BRINDA regression correction approach [5,6].

7 VA supplementation received by the child in the past 6 mo, as reported in the survey.

### Associations between BMI or BAZ, VA, and inflammation

**FIGURE 1:**
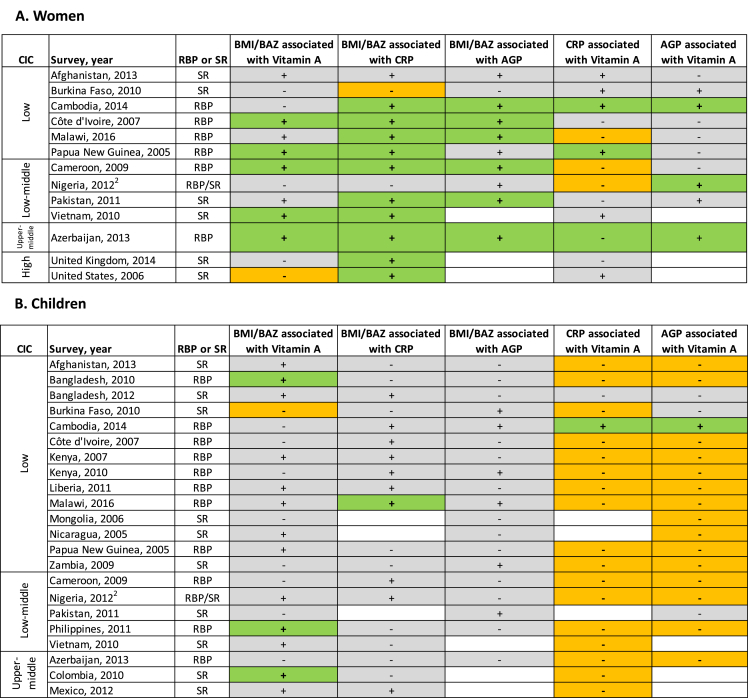
Patterns of association for vitamin A (RBP or SR), BMI or BAZ, and inflammation biomarkers (CRP or AGP) among women (A) and children (B) in 24 surveys: BRINDA project. Associations from adjusted linear regression models are presented. Green cells with a **+** indicate a significant positive association; orange cells with a − indicate a significant negative association (*P* < 0.05). Grey cells with + or − indicate the direction of nonsignificant associations. Blank cells indicate the variable was not available in that data set. See Supplemental Table 6 (women) and Supplemental Table 7 (children) for regression estimates for both unadjusted and adjusted models. All estimates accounted for the complex survey design (cluster, strata) with survey weights applied, except the Mongolia survey, which used simple random sampling. Vitamin A (RBP or SR) was measured in either serum or plasma, as reported by the survey. Covariates available for adjustment were age, education level (respondent, maternal, or household head), household socioeconomic status, access to an improved water source, access to an improved toilet, and urban/rural residence. Covariates were included in adjusted regression models if they were associated with the outcome variable at *P* < 0.1 in the unadjusted model. Inclusion criteria for analysis were BMI ≥18.5 (women), BAZ ≥−2 SD, or WHZ ≥−2 SD (children), not pregnant (women), and a negative malaria test result (both) to reduce potential confounding from inflammation due to illness or infection. CIC was defined according to the World Bank definition for the year in which the survey was conducted [35]. In the Nigeria survey for women, RBP was used for analysis as there were fewer missing values; for the children's survey from Nigeria, SR was used for analysis as there were fewer missing values. AGP, α1 acid glycoprotein; BAZ, BMI-for-age *z*-score; BRINDA, Biomarkers Reflecting Inflammation and Nutritional Determinants of Anemia; CIC, country income classification; CRP, C-reactive protein; RBP, retinol-binding protein; SR, serum retinol; WHZ, weight-for-height z-score.

Supplemental Table 6 contains women’s results from adjusted linear models that examined the relationship between BMI and VA biomarkers (retinol or RBP) and BMI and inflammation biomarkers (CRP and/or AGP). Overall, results were similar across CICs and most surveys (Figure 1).

In 5 of 13 women’s surveys, BMI was significantly positively correlated with retinol or RBP concentration (each *P* < 0.050: Côte d’Ivoire, Papua New Guinea, Cameroon, Vietnam, and Azerbaijan) (Figure 1). For each 1-unit increase in BMI, the retinol or RBP concentration increase ranged from 0.3% (Vietnam) to 1.0% (Papua New Guinea and Azerbaijan) (Supplemental Table 6). However, in the US survey, BMI was significantly negatively correlated with retinol concentration (−0.4%; 95% CI: −0.6%, −0.1%) (Supplemental Table 6).

BMI was significantly and positively associated with inflammation (CRP and/or AGP) in 10 of 13 women’s surveys (Figure 1). In contrast, the strength and direction of the relationship between inflammation and retinol or RBP concentration was inconsistent across women’s surveys, as well as between inflammatory markers (Figure 1). For example, in the survey from Nigeria, CRP was negatively associated with RBP concentration, whereas the relationship with AGP was positively associated (both *P* < 0.050).

Supplemental Table 7 contains children’s results from adjusted linear models that examined the relationship between BMI and VA biomarkers (retinol or RBP) and BMI and inflammation biomarkers (CRP and/or AGP). Overall and across CICs, most relationships between BAZ and retinol or RBP, and BAZ and inflammation biomarkers, were nonsignificant with varied directions of associations in adjusted models (Figure 1).

Children’s surveys from Bangladesh (2010), the Philippines, and Colombia had significant positive associations between BAZ and retinol or RBP; for every 1-unit increase in BAZ, the retinol or RBP concentration increase ranged from 1.5% (Bangladesh, 2010) to 2.9% (Philippines) (Supplemental Table 7). However, the survey from Burkina Faso showed a significant negative association between BAZ and retinol concentration (−9.9%; 95% CI: −18.4%, −0.4%).

Only 1 children’s survey (Malawi) showed a significant (positive) relationship between BAZ and inflammation in the adjusted models. In contrast, retinol or RBP concentration was significantly negatively associated with inflammation (CRP and/or AGP) in 19 of 22 children’s surveys (Figure 1).

### Mediation analysis among BMI, VA, and inflammation

**FIGURE 2:**
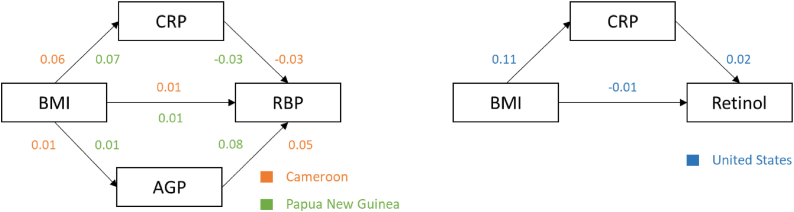
Examples of inconsistent mediation present in the women’s surveys from Cameroon, Papua New Guinea, and the United States: BRINDA project. The model for mediation analysis was *ln*Retinol/RBP = *β*_0_ + *β*_1_(BMI) + *M*_1_(*ln*CRP) [+*M*_2_(*ln*AGP)], where all values were continuous and AGP was included as a mediator only in analyses for which it was available in the data set (Cameroon and Papua New Guinea). Mediation was considered present when both the total and indirect effects were significant at *P* < 0.05 (Table 3) [38,39]. Inconsistent mediation is defined as the indirect and direct effects having opposite directions of associations [41]. Recognizing that models cannot infer causality, the simplified interpretation is as follows: total effect = the effect of BMI on retinol/RBP (value between BMI and retinol/RBP); direct effect = the effect of BMI on retinol/RBP controlling for inflammation (value not shown); indirect effect = the effect of BMI on retinol/RBP as mediated by the effect of CRP or AGP (values among BMI, CRP/AGP, and retinol/RBP). Indirect effects are calculated by multiplying the values between the independent variable (BMI) and the mediator (CRP) by the value between the mediator (CRP) and the dependent variable (retinol/RBP) and then summing that value with the second indirect effect when there is a second mediator (AGP) (Supplemental Figure 1). Covariates included in the specified models were age and education level (Cameroon); age, education level, and SES (Papua New Guinea); and age and SES (United States). Age was defined in years, education level as the highest level completed by the respondent, and SES was a 3-level ordinal variable of low, medium and high SES from the harmonized BRINDA project data set [23]. Covariates were included in the mediation model if they were associated with the outcome variable at *P* < 0.1 in bivariate models (Supplemental Table 6). AGP, α1 acid glycoprotein; BRINDA, Biomarkers Reflecting Inflammation and Nutritional Determinants of Anemia; CRP, C-reactive protein; ln, natural log; RBP, retinol-binding protein; SES, socioeconomic status.

Across surveys of both women and children, mediation findings were mostly nonsignificant, small in magnitude, and without consistent patterns by the CIC, or prevalence of overweight or obesity, inflammation, or VA insufficiency or deficiency. Among women, inflammation partially mediated the relationship between BMI and retinol or RBP in 3 of 13 surveys in adjusted models: Papua New Guinea, Cameroon, and the United States (Figure 2, Table 3). In all 3 surveys, the mediation was inconsistent, meaning that the direct and indirect effects operated in opposite directions [41]. In Papua New Guinea (with AGP) and the United States (with CRP), VA biomarkers were positively associated with inflammation, whereas RBP was negatively associated with CRP in both Cameroon and Papa New Guinea. In the survey from the United States, the percent change in retinol concentration mediated by CRP was 0.2% (95% CI: 0.1%, 0.4%) for every 1-unit increase in BMI (i.e., the indirect effect), and CRP mediated −69.2% of the relationship between BMI and retinol (i.e., CRP reduced the association between BMI and retinol by 69.2%) (Table 3). In both Papua New Guinea and Cameroon, RBP concentration, when mediated by AGP and CRP, changed by −0.2% for every 1-unit increase in BMI (indirect effect), and inflammation mediated −19.0% and −17.8%, respectively, of the relationship between BMI and RBP (Table 3). Moreover, compared with AGP, CRP mediated the majority of the proportion of total inflammation (Papua New Guinea: 77.0% CRP compared with 21.0% AGP; Cameroon: 82.6% CRP compared with 17.4% AGP); the survey from the United States did not measure AGP (Table 3). Post hoc mediation analyses that additionally adjusted for SES (conducted in all surveys where this covariate was available but not included in the primary mediation models because it was nonsignificant in bivariate analyses) did not change the final mediation results.

**TABLE 3:** Relationships between VA (RBP or SR) and BMI as mediated by inflammation among women (15–49 y) with normal weight to overweight/obesity by survey: BRINDA project^1^.

CIC^2^	Country, survey year	*n*	RBP or SR	Women’s mediation analysis, adjusted^3^					
				Total effect	Direct effect	Indirect effect	% Mediated	% Mediated by CRP	% Mediated by AGP
Low	Afghanistan, 2013	570	SR	0.2 (−1.3, 1.8)	0.1 (−1.5, 1.8)	0.1 (−0.1, 0.2)	NM	—	—
	Burkina Faso, 2010	61	SR	−0.2 (−4.5, 4.4)	−0.03 (−5.5, 5.7)	−0.1 (−2.7, 2.4)	NM	—	—
	Cambodia, 2014	609	RBP	−0.9 (−2.2, 0.4)	−2.4 (−3.6, −1.1)	1.5 (0.2, 2.8)	NM	—	—
	Côte d’Ivoire, 2007	706	RBP	0.7 (0.2, 1.3)	0.9 (0.3, 1.5)	−0.2 (−0.3, 0.0003)	NM	—	—
	Malawi, 2016	595	RBP	0.9 (−0.1, 1.9)	1.2 (−0.04, 2.4)	−0.3 (−0.5, 1.3)	NM	—	—
	Papua New Guinea, 2005	658	RBP	1.0 (0.3, 1.7)	1.2 (0.4, 1.9)	−0.2 (−0.4, −0.01)	−19.0	77.0	21.0
Low-middle	Cameroon, 2009	554	RBP	0.8 (0.3, 1.4)	1.0 (0.4, 1.5)	−0.2 (−0.3, −0.03)	−17.8	82.6	17.4
	Nigeria, 2012	506	RBP	−0.1 (−0.7, 0.5)	−0.1 (−0.7, 0.5)	0.1 (−0.2, 0.3)	NM	—	—
	Pakistan, 2011	4851	SR	0.3 (−0.3, 0.8)	0.3 (−0.3, 0.8)	−0.02 (−0.1, 0.1)	NM	—	—
	Vietnam, 2010	1138	SR	0.9 (−0.03, 1.9)	0.9 (−0.2, 1.9)	0.1 (−0.3, 0.5)	NM	—	—
Upper-middle	Azerbaijan, 2013	2506	RBP	1.0 (0.7, 1.2)	0.9 (0.7, 1.2)	0.1 (−0.1, 0.2)	NM	—	—
High	United Kingdom, 2014	568	SR	−0.3 (−0.6, −0.1)	−0.3 (−0.9, 0.3)	−0.03 (−0.3, 0.3)	NM	—	—
	United States, 2006	2847	SR	−0.3 (−0.5, −0.1)	−0.6 (−0.8, −0.3)	0.2 (0.1, 0.4)	−69.2	100	—

Abbreviations: AGP, α1-acid glycoprotein; BRINDA, Biomarkers Reflecting Inflammation and Nutritional Determinants of Anemia; CIC, country income classification; CRP, C-reactive protein; NM, no mediation; RBP, retinol-binding protein; SR, serum or plasma retinol; VA, vitamin A.

1 RBP, SR, CRP, and AGP variables were natural log transformed for analysis due to nonnormal distributions. Mediation estimates were exponentiated, and results are presented as percent change (95% CI) in VA (RBP or SR) concentration for every 1-unit change in BMI, adjusted for available covariates. RBP or SR concentration measured in serum or plasma, as reported in the survey. All estimates account for cluster survey design (cluster and strata), with survey weights applied. Inclusion criteria were BMI ≥ 18.5, not pregnant, and a negative malaria test result to reduce potential confounding from inflammation due to illness or infection.

2 CIC defined according to the World Bank definition for the year in which the survey was conducted [35].

3 Model for mediation analysis: *ln*Vitamin A [RBP or SR] = *β*_0_ + *β*_1_(BMI) + *M*_1_(*ln*CRP) [+*M*_2_(*ln*AGP)], where all values were continuous and AGP was included as a mediator only in analyses for which it was available in the data set. Recognizing that models cannot infer causality, the simplified interpretation is as follows: total effect = the effect of BMI on VA (retinol or RBP) not controlling for inflammation; direct effect = the effect of BMI on VA (retinol or RBP) controlling for inflammation; and indirect effect = the effect of BMI on VA (retinol or RBP) mediated by the effect of CRP and/or AGP. Mediation was considered present when both the total and indirect effects were significant [38,39]. Covariates available for adjustment were age, education level (respondent or household head), household socioeconomic status, access to an improved water source, access to an improved toilet, and urban/rural residence. Covariates were included in the mediation model if they were associated with the outcome variable at *P* < 0.1 in bivariate models (Supplemental Table 6). Unadjusted mediation estimates are presented in Supplemental Table 8.

Among children, mediation was present in 1 (Philippines) of 22 surveys; however, the significance was marginal (*P* = 0.050 for the indirect effect) (Table 4). In this survey, inflammation positively mediated 24.3% of the relationship between BAZ and RBP, a 0.8% increase in RBP concentration for every 1-unit change in BAZ (Table 4). When compared with AGP, CRP mediated the majority of the proportion of total inflammation (89.6% CRP compared with 9.1% AGP) (Table 4). Unadjusted mediation results for both women and children are presented in Supplemental Table 8.

**TABLE 4:** Relationships between VA (retinol or RBP) and BAZ as mediated by inflammation among children (6–59 mo) with normal weight to overweight/obesity by survey: BRINDA project^1^.

CIC^2^	Country, survey year	*n*	RBP or SR	Children’s mediation analysis, adjusted^3^					
				Total effect	Direct effect	Indirect effect	% Mediated	% Mediated by CRP	% Mediated by AGP
Low	Afghanistan, 2013	356	SR	1.4 (−3.0, 5.7)	−0.3 (−4.0, 3.4)	1.7 (−0.3, 3.7)	NM	—	—
	Bangladesh, 2010	1165	RBP	1.6 (0.5, 2.5)	1.3 (0.4, 2.1)	0.3 (−0.3, 0.8)	NM	—	—
	Bangladesh, 2012	303	SR	3.6 (−0.3, 7.3)	3.8 (0.2, 7.3)	−0.2 (−1.0, 0.5)	NM	—	—
	Burkina Faso, 2010	60	SR	−9.9 (−18.5, −2.3)	−10.7 (−19.7, −3.0)	1.0 (−4.3, 6.2)	NM	—	—
	Cambodia, 2014	599	RBP	−2.0 (−8.5, 4.4)	−1.5 (−4.6, 1.5)	−0.5 (−5.7, 4.6)	NM	—	—
	Côte d’Ivoire, 2007	432	RBP	−0.2 (−2.9, 2.5)	0.3 (−1.8, 2.4)	−0.5 (−1.8, 0.7)	NM	—	—
	Kenya, 2007	624	RBP	0.9 (−2.0, 3.7)	1.0 (−1.6, 3.7)	−0.2 (−1.0, 0.6)	NM	—	—
	Kenya, 2010	525	RBP	−2.0 (−4.8, 0.9)	−1.3 (−4.0, 1.4)	−0.7 (−1.8, 0.5)	NM	—	—
	Liberia, 2011	929	RBP	1.9 (−0.1, 3.8)	1.9 (0.03, 3.8)	−0.1 (−0.7, 0.6)	NM	—	—
	Malawi, 2016	746	RBP	1.5 (−1.1, 4.1)	2.8 (0.4, 5.0)	−1.3 (−2.3, −0.3)	NM	—	—
	Mongolia, 2006	202	SR	−0.9 (−9.2, 7.5)	−1.6 (−9.9, 6.6)	0.8 (−0.7, 2.2)	NM	—	—
	Nicaragua, 2005	1403	SR	0.9 (−1.8, 3.5)	0.8 (−2.0, 3.5)	0.1 (−0.3, 0.5)	NM	—	—
	Papua New Guinea	369	RBP	2.5 (0.1, 4.7)	1.7 (−0.5, 3.9)	0.7 (−0.2, 1.7)	NM	—	—
	Zambia, 2009	313	SR	0.03 (−3.3, 3.4)	−0.02 (−3.4, 3.4)	0.1 (−1.1, 1.2)	NM	—	—
Low-middle	Cameroon, 2009	546	RBP	−1.9 (−4.2, 0.3)	−1.5 (−3.6, 0.6)	−0.5 (−1.3, 0.4)	NM	—	—
	Nigeria, 2012	283	SR	1.6 (−1.3, 4.4)	1.9 (−0.8, 4.6)	−0.3 (−1.3, 0.6)	NM	—	—
	Pakistan, 2011	5003	SR	−1.6 (−3.6, 0.4)	−1.6 (−3.6, 0.4)	−0.02 (−0.1, 0.1)	NM	—	—
	Philippines, 2011	1503	RBP	3.1 (1.2, 4.9)	2.4 (0.5, 4.2)	0.8 (−0.01, 1.5)^4^	24.3	89.6	9.1
	Vietnam, 2010	329	SR	2.6 (−1.0, 6.1)	2.2 (−1.3, 5.7)	0.4 (−0.2, 1.0)	NM	—	—
Upper-middle	Azerbaijan, 2013	986	RBP	−0.1 (−1.9, 1.6)	−0.8 (−2.2, 0.7)	0.7 (−0.1, 1.4)	NM	—	—
	Colombia, 2010	3711	SR	2.2 (0.7, 3.6)	2.1 (0.6, 3.5)	0.1 (−0.1, 0.3)	NM	—	—
	Mexico, 2012	2430	SR	1.3 (−0.4, 2.9)	1.7 (0.03, 3.4)	−0.5 (−1.1, 0.2)	NM	—	—

Abbreviations: AGP, α1-acid glycoprotein; BAZ, BMI-for-age *z*-score; BRINDA, Biomarkers Reflecting Inflammation and Nutritional Determinants of Anemia; CIC, country income classification; CRP, C-reactive protein; NM, no mediation; RBP, retinol-binding protein; SR, serum or plasma retinol; VA, vitamin A.

1 SR, RBP, CRP, and AGP variables were natural log transformed for analysis due to nonnormal distributions. Mediation estimates were exponentiated, and results are presented as percent change (95% CI) in VA (SR or RBP) concentration for every 1-unit change in BAZ, adjusted for available covariates. SR or RBP concentration measured in serum or plasma, as reported in the survey. All estimates account for cluster survey design (cluster and strata) with survey weights applied, except in the survey from Mongolia, which used simple random sampling. Inclusion criteria were BAZ or WHZ ≥−2 SD and a negative malaria test result to reduce potential confounding from inflammation due to illness or infection.

2 CIC defined according to the World Bank definition for the year in which the survey was conducted [35].

3 Model for mediation analysis: *ln*Vitamin A [RBP or SR] = *β*_0_ + *β*_1_(BAZ) + *M*_1_(*ln*CRP) [+*M*_2_(*ln*AGP)], where all values were continuous and CRP or AGP were included as a mediator only in analyses for which it was available in the data set. Recognizing that models cannot infer causality, the simplified interpretation is as follows: total effect = the effect of BAZ on VA (retinol or RBP) not controlling for inflammation; direct effect = the effect of BAZ on VA (retinol or RBP) controlling for inflammation; and indirect effect = the effect of BAZ on VA (retinol or RBP) mediated by the effect of CRP and/or AGP. Mediation was considered present when both the total and indirect effects were significant [38,39]. Covariates available for adjustment were age, education level (head of household or maternal education level), household socioeconomic status, access to an improved water source, access to an improved toilet, and urban/rural residence. Covariates were included in the mediation model if they were associated with the outcome variable at *P* < 0.1 in bivariate models (Supplemental Table 7). Unadjusted mediation estimates are presented in Supplemental Table 8.

4 *P* value for indirect effect = 0.05, marginally significant.

### Sensitivity analyses

Among surveys that measured malaria status (*n* = 4 women; *n* = 8 children), results from the sensitivity mediation analysis that included all observations regardless of malaria test result were similar to the main mediation results that excluded observations with a positive malaria test result (Supplemental Table 9). Results from the sensitivity analysis among surveys that additionally excluded observations with morbidity data of reported fever or diarrhea were also similar to the main mediation results (Supplemental Table 10). In a sensitivity analysis of surveys that measured both retinol and RBP (*n* = 4 women; *n* = 4 children), there were no substantive changes in results (Supplemental Table 11). However, in a small subsample in the women’s survey from Malawi (retinol subsample, *n* = 65; RBP full sample, *n* = 595), there appeared to be a small (3.8% increase) but significant (*P* = 0.010) relationship between BMI and retinol. This relationship was completely mediated through CRP. Conversely, in the women’s survey from Cameroon, the significant relationship that was present between BMI and RBP in the full sample (*n* = 594) became nonsignificant in the subsample analysis with retinol (*n* = 82) (Supplemental Table 11).

## Discussion

In analyses of 24 national and regional survey data sets, we found that inflammation (measured by CRP and AGP) was generally not a statistical mediator of the association of overweight and obesity with VA status (measured by retinol or RBP). We observed mediation in only 4 surveys and with effect sizes of <1%. Although inflammation associated with overweight or obesity could theoretically affect VA biomarker interpretation, our findings suggest that it is unlikely to be an important consideration in population VA surveillance. We considered that these results might vary by country income category, reflecting differences in mean BMI, but no patterns by World Bank income category emerged in mediation analyses. However, the consistent negative associations between CRP and AGP concentrations and VA biomarkers among children reinforce current recommendations to measure inflammation biomarkers (regardless of etiology) to interpret VA status.

Despite minimal mediation effects, these findings address an important methodological gap. Prior BRINDA project analyses aimed to characterize the associations between inflammation biomarkers and micronutrient biomarkers and define corresponding adjustment methods [5,6,20], but they did not distinguish whether null associations between inflammation and VA biomarkers in women reflected true biology or confounding by adiposity-related inflammation. Our study differs from prior analyses in that we examined associations with weight status and applied a mediation framework to statistically quantify the extent to which inflammation explains the association between BMI and retinol or RBP. By demonstrating that obesity-associated inflammation did not meaningfully mediate relationships between BMI and VA biomarkers, we confirm that current population assessment protocols do not require special adjustment for adiposity-related inflammation among women or children, helping to resolve uncertainty for surveillance programs in contexts characterized by elevated BMI and lower burden of undernutrition and infectious illness, contexts that are common in countries experiencing nutrition transition. Considering this purpose, this analysis applied more restrictive exclusion criteria than prior BRINDA project analyses, excluding individuals with BMI of <18.5 (women), wasting (children), and malaria. The results thus do not reflect contexts with high prevalence of undernutrition.

Despite the difference in eligibility criteria, the results are consistent with observations from previous BRINDA project analyses in that inflammation was not consistently associated with VA biomarkers among women but that, among children, inflammation biomarkers were strongly and consistently associated with biomarkers of VA [5,6,20]. Therefore, the findings support the existing recommendations to assess inflammation, regardless of etiology, when assessing VA status of children [5,6,20]. Among women, although our findings identified BMI as a correlate of inflammation in many surveys, these associations did not change the conclusion that inflammation had weak and inconsistent relationships with VA biomarkers, suggesting that adjustment for inflammation may not consistently improve estimates of VA deficiency prevalence in this group.

With mediation analysis, we observed that inflammation weakened the association between BMI and VA biomarkers in 2 of 13 women’s surveys (Cameroon, Papua New Guinea) but that this relationship was strengthened in the survey from the United States. Given that the mediation effects were inconsistent (i.e., the direct and indirect effects had opposite signs) [41] and the magnitude of association was small across all 3 surveys (i.e., the percent change in retinol or RBP concentration for every 1-unit increase in BMI ranged from −0.2% to 0.2%), these suppression effects likely reflect weak and variable relationships among BMI, inflammation, and VA biomarkers across surveys. The associations between inflammation and VA biomarkers were also not consistent with expected biological pathways in Papua New Guinea and the United States, as inflammation is known to decrease circulating retinol and RBP. Considering that most of the mediation results were not significant, the few significant mediation results may be statistical artifacts with minimal implications for population-level VA assessment.

This study was motivated by growing literature on RBP concentrations in the context of obesity and noncommunicable disease. In women’s adjusted linear regression analyses, we found that BMI was generally positively associated with retinol or RBP. Although circulating RBP concentrations (and by association, retinol) appear primarily derived from the liver [10,42,43], RBP (or RBP4) is also expressed in adipose tissue and has been identified as an adipokine [10,44]. Because of its role as an adipokine, circulating RBP has been associated with obesity and noncommunicable disease in adults [[7], [8], [9], [10], [11],^17^,^44^,^45^], and in children and adolescents [9,46,47], although the direction and magnitude of associations vary and causal pathways remain unclear. However, substantial gaps remain in this literature, particularly regarding the role of adipocyte-derived RBP in states of chronic disease and links with inflammatory markers [[7], [8], [9], [10],^48^,^49^]. Although current evidence supports a negative relationship between inflammation and circulating RBP (and retinol) concentrations, which may complicate VA status assessment, additional information is needed to clarify relationships between chronic disease and RBP and/or retinol, including establishing temporal relationships and measurement of VA stores. Given rising global prevalence of overweight and obesity, including among children [50], better understanding of how adiposity impacts VA metabolism and assessment would be valuable.

Among women, CRP and/or AGP were consistently positively associated with BMI, which aligns with the literature that inflammation is often elevated in overweight and obesity [12,13,51,52]. Although both inflammatory biomarkers correlate with BMI, the literature is more extensive for CRP [[53], [54], [55], [56], [57], [58]] than AGP [59,60], even though AGP is generally thought of as a longer-term inflammatory biomarker [61]. Prior studies also suggest CRP may be more strongly associated with retinol and RBP than AGP [[62], [63], [64]]. As CRP and AGP are often the only inflammatory biomarkers available for measurement in epidemiological surveys, incorporating a broader range of acute-phase proteins would help understand which markers could best inform micronutrient assessment.

Among children, inflammation was significantly associated with lower retinol or RBP in all but 3 surveys (Bangladesh 2012, Cameroon, and Pakistan). We speculate that the inflammation driving these associations may be due to helminth or parasitic infections, upper respiratory infections, or other common inflammatory illnesses among children that were not part of our exclusion criteria but have been found to be associated in other BRINDA project analyses [40,52]. Although elevated CRP is associated with elevated BMI in studies among children and adolescents [55,65], the low median BAZ (SD: 0.2) among children in our analyses may explain why we observed few associations between BAZ and inflammation and between BAZ and retinol or RBP.

Strengths of this study include the development of our analytical plan and exclusion criteria prior to completing any analyses. Moreover, the surveys included in our analyses represented a diversity of geographic regions and CICs, although survey inclusion was limited to BRINDA project data sets with available VA and inflammation biomarker and anthropometric data, resulting in uneven global representation.

Our study has a number of limitations. Because these analyses were cross-sectional, we were unable to explore temporal relationships between changes in BMI, inflammation, and VA biomarkers, and mediation findings should be interpreted as statistical associations only. The eligibility criteria may limit the generalizability of the results. Because the analytic sample was restricted to observations with normal weight to overweight/obesity and excluded those with malaria or acute illness, >70% of observations were excluded in the surveys from Afghanistan (women and children), Burkina Faso (women and children), Mexico (children), and Pakistan (women), and >60% of the analytical sample was from high SES households in the majority of surveys. Sensitivity analyses that included the observations excluded for malaria or morbidity symptoms (e.g., fever and diarrhea) did not change the main results, and we based the conclusions on the full set of results, the majority of which were from surveys with >500 observations, to avoid putting undue influence on these surveys. Nevertheless, our findings have limited generalizability to populations with high burdens of undernutrition or infection. The exclusion of underweight or wasted individuals and malaria-positive cases means our null mediation results are expected to reflect contexts where obesity-related inflammation predominates over infection-related inflammation. In populations with high infectious disease burden, inflammation from infections (rather than adiposity) likely remains the primary driver of VA biomarker suppression, and existing BRINDA project adjustment protocols remain appropriate. Our findings specifically address the question of whether adiposity adds independent confounding beyond infectious inflammation.

Additionally, in 3 surveys (Afghanistan, Colombia, and Zambia), we generated and imputed random CRP values between 0 and the lowest CRP value as >30% of the analytical sample had a single value for the lower limit of detection. These limitations of the data may introduce bias or obscure real associations; however, we confirmed that the direction and strength of association did not differ with the randomly generated values by reanalyzing all models with multiple random seeds. In addition, no significant mediation was observed in any of these 3 surveys, and excluding them from review of the survey-specific results does not change the study conclusions.

Our analyses were also limited by the selected measurement parameters, which likely influenced our findings. For example, overweight and obesity were defined using BMI among adults, which does not directly measure adiposity nor metabolically active adipose tissue, although BMI has been found to be highly correlated with waist circumference and predictive of cardiometabolic risk [66], supporting its use for surveillance programs and comparative analyses. Additionally, although BAZ is not recommended for use with children aged <24 mo [67], our use of BAZ was based on prior BRINDA project analyses where use of BAZ rather than weight-for-height *z*-score did not affect the conclusions [21]. These measurement limitations should be considered when interpreting our findings as they may introduce measurement imprecision, particularly among younger children, but we believe their use was unlikely to change the overall direction or strength of observed associations. In these data sets, measurement of inflammation was limited to CRP and AGP, which inadequately represent all adipose-derived inflammatory markers. Although there were no other inflammatory biomarkers available for analysis, many epidemiological studies measure inflammation with CRP and AGP and use them for interpreting retinol and RBP concentrations; thus, their inclusion in this study may enhance the comparability of our findings with other studies. It should be noted that 5 surveys did not contain a variable to indicate pregnancy, and in the remaining surveys, pregnancy status was self-reported. Thus, our results may include data from pregnant women, which may have introduced error into our results and biased associations toward the null considering that pregnancy is an inflammatory state that may alter VA metabolism [68]. Moreover, dietary data were not available to understand the relative contributions of different sources of VA or whether intake modifies the observed associations. Instead, we focused on relationships between biomarkers.

The differing laboratory practices and calibration techniques used by laboratories involved in sample analyses across the 24 individual surveys may have introduced random or systematic error, affecting our biomarker interpretation [36,69]. However, the fact that we observed consistent relationships between BMI and inflammation for women and for inflammation and VA biomarkers for children, suggest that the null results observed elsewhere were not solely attributable to variation in laboratory methods. Alternatively, we cannot rule out the influence of residual confounding or the possibility of chance findings in the observed results. Finally, retinol and RBP represent circulating transport proteins that do not directly reflect VA total body stores due to homeostatic regulation [3]. Although retinol and RBP concentrations can identify when liver VA stores are very low, they are not good individual level biomarkers, and their utility for population assessment has been questioned [70]. Nevertheless, these biomarkers represent the most commonly measured biomarkers for population assessment [2,31] and are useful to examine with respect to methodological factors that may affect their interpretation. Although we assumed an imprecise biological 1:1 ratio of retinol to RBP [71] for descriptive analyses and, in sensitivity, analyses we did see some inconsistencies in the relationship between BMI and retinol or RBP when measured in the same survey, these results do not change our conclusions.

In conclusion, research on population VA assessment has largely concentrated on contexts where undernutrition is common, whereas the role of inflammation associated with overweight and obesity in VA assessment has been less studied. Our findings suggest that, among women and children, inflammation associated with overweight and obesity specifically is unlikely to influence VA assessment when measured with retinol or RBP. We also confirmed prior findings that inflammation (regardless of etiology) consistently impacts VA assessment with retinol and RBP among children and inconsistently among women. Because of the limitations for individual and clinical assessment, this guidance should be applied only in the context of population assessments. When planning and implementing population VA surveillance, measuring biomarkers of inflammation, especially among children, and considering contextual factors such as possible sources of inflammation and prevalence of common health conditions, may improve interpretation of results.

## Author contributions

The author responsibilities’ were as follows—AW, MY, PS, RES: designed the research; JND: performed statistical analyses and wrote the paper; RES: responsible for the final content; and all authors: contributed to interpretation of the results, revision of the manuscript, and read and approved the final manuscript.

## Data availability

Data described in the manuscript, code book, and analytic code will be made available upon request pending approval from the Biomarkers Reflecting Inflammation and Nutritional Determinants of Anemia steering committee and country representatives.

## Disclaimers

The findings and conclusions in this article are those of the authors and do not necessarily represent the official position of the US Centers for Disease Control and Prevention.

## Declaration of Generative AI and AI-assisted technologies in the writing process

The authors declare that no generative AI or AI-assisted technologies were used in the writing of this manuscript.

## Funding

The Biomarkers Reflecting Inflammation and Nutritional Determinants of Anemia project was supported by the Bill & Melinda Gates Foundation, Centers for Disease Control and Prevention, Eunice Kennedy Shriver National Institute of Child Health and Human Development, HarvestPlus, and the United States Agency for International Development. This analysis was also supported in part by USDA Hatch project CA-D-NTR-2391-H (RES).

## Conflict of interest

RE-S is an Editor for *Current Developments in Nutrition* and played no role in the Journal’s evaluation of the manuscript. The other authors report no conflicts of interest.
