# Supplementary material for: Inflammation Does Not Mediate Relationships between BMI/BMI-for-Age Z-Score and Retinol or Retinol-Binding Protein in Adult Women or Young Children without Underweight, Wasting, or Malaria: An Analysis of 24 Surveys
Source: Curr Dev Nutr. 2026 Apr 2;10(5):107684. doi: 10.1016/j.cdnut.2026.107684 (PMC13133937; doi:10.1016/j.cdnut.2026.107684)
Supplement: multimedia component 1 [file mmc1.docx]

**SUPPLEMENTAL MATERIAL for:** Inflammation does not mediate relationships between BMI/BMI-for-age z-score and retinol or retinol-binding protein among adult women or young children without underweight, wasting, or malaria: an analysis of 24 surveys by Davis et al.

1. **Supplemental Figure 1.** Mediation analysis model
2. **Supplemental Table 1.** Number and proportion of observations excluded due to exclusion criteria for women, by survey: BRINDA project
3. **Supplemental Table 2.** Number and proportion of observations excluded due to exclusion criteria for children, by survey: BRINDA project
4. **Supplemental Table 3.** Available covariates in each survey: BRINDA project
5. **Supplemental Table 4.** Age and household characteristics for women (15-49 years) with normal weight to overweight/obesity by survey: BRINDA project
6. **Supplemental Table 5.** Age, sex, and household characteristics for children (6-59 months) with normal weight to overweight/obesity by survey: BRINDA project
7. **Supplemental Table 6.** Bivariate (B, unadjusted) and multivariable (M, adjusted) percent change associations between Vitamin A, CRP, AGP, and BMI among women (15-49 years) with normal weight to overweight/obesity by survey: BRINDA project
8. **Supplemental Table 7.** Bivariate (B, unadjusted) and multivariable (M, adjusted) percent change associations between Vitamin A, CRP, AGP, and BAZ among children (6-59 months) with normal weight to overweight/obesity by survey: BRINDA project
9. **Supplemental Table 8.** Unadjusted relationships between vitamin A (retinol or RBP) and BMI or BAZ as mediated by inflammation among women (15-49 years) and children (6-59 months) with normal weight to overweight/obesity by survey: BRINDA project
10. **Supplemental Table 9.** Malaria sensitivity analysis: mediation analysis (unadjusted) assessing the relationship between Vitamin A (RBP or SR), BMI or BAZ and inflammation including and excluding observations that tested positive for malaria for women and children with normal weight to overweight/obesity: BRINDA project
11. **Supplemental Table 10.** Morbidity sensitivity analysis: mediation analysis (unadjusted) assessing the relationship between Vitamin A (RBP or SR), BMI or BAZ and inflammation excluding and including observations that reported fever and/or diarrhea in the past 2 weeks (women) and in the past 24 hours (children) with normal weight to overweight/obesity: BRINDA project
12. **Supplemental Table 11.** Sensitivity mediation analysis examining relationships among both biomarkers of vitamin A (retinol binding protein or serum retinol) and BMI as mediated by inflammation among women (15-49 years) and children (6-59 months) with normal weight to overweight/obesity by survey: BRINDA project
13. **Supplemental References**

**Supplemental Figure 1.** Mediation analysis model


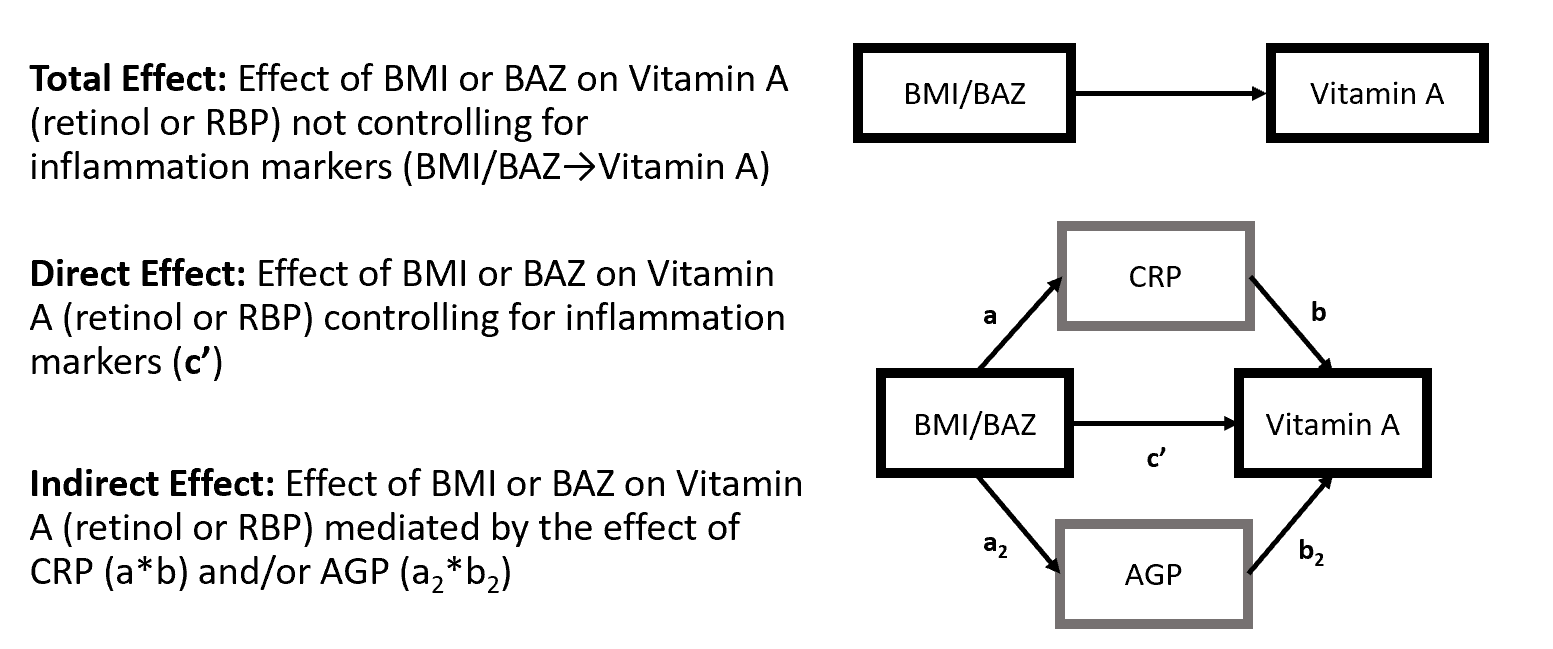


**Supplemental Figure 1 Footnote.** In surveys without CRP or AGP variables, the mediation analysis path was eliminated (1,2). Abbreviations: AGP, alpha-1 acid glycoprotein; BAZ, BMI-for-age z-score; CRP, C-reactive protein; RBP, retinol-binding protein.

**Supplemental Table 1.** Number and proportion of observations excluded due to exclusion criteria for women, by survey: BRINDA project^1^

|  | | **Exclusion Criteria** | | | | | | | | |  | |
| --- | --- | --- | --- | --- | --- | --- | --- | --- | --- | --- | --- | --- |
| **Survey, year** | **Total obs. in dataset** | **BMI <18.5 kg/m^2^** | **Implausible Height** | **Implausible Weight** | **Pregnant** | **Positive Malaria Result** | **Missing BMI** | **Missing SR or RBP** | **Missing CRP** | **Missing AGP** | **Total obs. excluded** | **Analytical sample size & proportion of total** |
|  | n | n (%) | n (%) | n (%) | n (%) | n (%) | n (%) | n (%) | n (%) | n (%) | n (%) | n (%) |
| Afghanistan, 2013 | 23875 | 954 (4.0) | 0 (0) | 0 (0) | 0 (0) | -- | 13991 (58.6) | 22829 (95.6) | 22825 (95.6) | 22825 (95.6) | 23304 (97.6) | 571 (2.4) |
| Burkina Faso, 2010 | 484 | 60 (12.4) | 0 (0) | 0 (0) | 0 (0) | 36 (7.4) | 70 (14.5) | 340 (70.2) | 355 (73.3) | 355 (73.3) | 423 (87.4) | 61 (12.6) |
| Cambodia, 2014 | 724 | 94 (13.0) | 2 (0.3) | 1 (0.1) | 0 (0) | -- | 0 (0) | 19 (2.6) | 19 (2.6) | 19 (2.6) | 115 (15.9) | 609 (84.1) |
| Côte D'Ivoire, 2007 | 863 | 81 (9.4) | 11 (1.3) | 2 (0.2) | -- | 39 (4.5) | 14 (1.6) | 29 (3.4) | 29 (3.4) | 29 (3.4) | 157 (18.2) | 706 (81.8) |
| Malawi, 2016 | 804 | 70 (8.7) | 1 (0.1) | 0 (0) | 0 (0) | 116 (14.4) | 17 (2.1) | 28 (3.5) | 28 (3.5) | 28 (3.5 | 210 (26.1) | 595 (74.0) |
| Papua New Guinea, 2005 | 779 | 51 (6.5) | 4 (0.5) | 0 (0) | -- | -- | 14 (1.8) | 30 (3.9) | 30 (3.9) | 30 (3.9) | 87 (11.2) | 692 (88.8) |
| Cameroon, 2009 | 787 | 67 (8.5) | 0 (0) | 0 (0) | -- | 108 (13.7) | 4 (0.5) | 27 (3.4) | 27 (3.4) | 27 (3.4) | 178 (22.6) | 594 (75.5) |
| Nigeria, 2012 | 620 | 55 (8.9) | 0 (0) | 1 (0.2) | -- | 56 (9.0) | 10 (1.6) | 0 (0) | 0 (0) | 0 (0) | 114 (18.4) | 506 (81.6) |
| Pakistan, 2011 | 22278 | 3024 (15.6) | 3 (0.01) | 4 (0.02) | 0 (0) | -- | 361 (1.6) | 14265 (64.0) | 14381 (64.6) | 14017 (63.0) | 17332 (77.8) | 4946 (22.2) |
| Vietnam, 2010 | 1492 | 305 (20.4) | 0 (0) | 0 (0) | -- | -- | 1 (0.1) | 52 (3.5) | 9 (0.6) | -- | 354 (23.7) | 1138 (76.3) |
| Azerbaijan, 2013 | 2910 | 138 (4.7) | 0 (0) | 0 (0) | 0 (0) | -- | 73 (2.5) | 254 (8.7) | 254 (8.5) | 254 (8.7) | 382 (13.1) | 2528 (86.9) |
| United Kingdom, 2014 | 2050 | 69 (3.7) | 0 (0) | 0 (0) | 0 (0) | -- | 93 (4.5) | 1153 (56.2) | 1108 (54.1) | -- | 1214 (59.2) | 836 (40.8) |
| United States, 2006 | 3456 | 143 (4.1) | 0 (0) | 1 (0.03) | 0 (0) | -- | 47 (1.4) | 311 (9.0) | 259 (7.5) | -- | 467 (13.5) | 2989 (86.5) |

^1^Exclusion criteria were: BMI <18.5 kg/m^2^; height or weight outside the ranges of 101.6-219.9 cm and 22.7-222.2 kg;(3) pregnant; positive malaria test result; or missing values for RBP, SR (missing reported only on the RBP or SR variable included in our primary analyses), CRP, AGP, or BMI (due to missing values for weight or height). Some observations may be excluded from multiple categories. Exclusion criteria percentages are proportions of total observations in the individual datasets. '--’ indicates the variable was not available in that survey. Abbreviations: AGP, α-1-acid glycoprotein; BRINDA, Biomarkers Reflecting Nutritional Determinants of Anemia; CRP, C-reactive protein; obs., observations; RBP, retinol-binding protein; SR, serum retinol.

**Supplemental Table 2.** Number and proportion of observations excluded due to exclusion criteria for children, by survey: BRINDA project^1^

|  |  | **Exclusion Criteria** | | | | | | | | |  |  |
| --- | --- | --- | --- | --- | --- | --- | --- | --- | --- | --- | --- | --- |
| **Survey, year** | **Total obs. in dataset** | **BAZ**  **<-2 SD** | **WHZ <-2 SD** | **Implausible BAZ** | **Implausible WHZ** | **Positive Malaria Result** | **Missing BAZ** | **Missing SR or RBP** | **Missing CRP** | **Missing AGP** | **Total obs. excluded** | **Analytical sample size & proportion of total** |
|  | n | n (%) | n (%) | n (%) | n (%) | n (%) | n (%) | n (%) | n (%) | n (%) | n (%) | n (%) |
| Afghanistan, 2013 | 19896 | 1598 (8.0) | 1823 (9.2) | 744 (3.8) | 424 (2.1) | -- | 341 (1.7) | 19239 (96.7) | 19231 (96.7) | n/a | 19310 (97.0) | 586 (3.0) |
| Bangladesh, 2010 | 1561 | 305 (19.5) | 277 (17.7) | 12 (0.8) | 8 (0.5) | -- | 18 (1.2) | 68 (4.4) | 68 (4.4) | 68 (4.4) | 382 (24.5) | 1179 (75.5) |
| Bangladesh, 2012 | 1108 | 134 (12.1) | 161 (14.5) | 26 (2.3) | 19 (1.7) | -- | 85 (7.7) | 235 (21.2) | 637 (57.5) | 637 (57.5) | 748 (67.5) | 360 (32.5) |
| Burkina Faso, 2010 | 482 | 5 (1.1) | 8 (1.7) | 0 (0) | 0 (0) | 9 (1.9) | 73 (15.1) | 357 (74.1) | 357 (74.1) | 357 (74.1) | 422 (87.6) | 60 (12.4) |
| Cambodia, 2014 | 874 | 60 (6.9) | 69 (7.9) | 5 (0.6) | 1 (0.1) | -- | 0 (0) | 209 (23.9) | 209 (23.9) | 209 (23.9) | 275 (31.5) | 599 (68.5) |
| Côte D'Ivoire, 2007 | 864 | 104 (12.0) | 116 (13.4) | 29 (3.4) | 16 (1.9) | 214 (24.8) | 37 (4.3) | 118 (13.7) | 118 (13.7) | 118 (13.7) | 429 (49.7) | 435 (50.3) |
| Kenya, 2007 | 1056 | 47 (4.5) | 51 (4.8) | 5 (0.5) | 5 (0.5) | 196 (18.6) | 40 (3.8) | 160 (15.2) | 160 (15.2) | 160 (15.2) | 391 (37.0) | 665 (63.0) |
| Kenya, 2010 | 896 | 26 (2.9) | 29 (3.2) | 4 (0.5) | 3 (0.3) | 276 (30.8) | 31 (3.5) | 47 (5.2) | 47 (5.3) | 47 (5.3) | 345 (38.5) | 551 (61.5) |
| Liberia, 2011 | 1476 | 129 (8.7) | 152 (10.3) | 3 (0.2) | 3 (0.2) | 358 (24.3) | 10 (0.7) | 42 (2.8) | 42 (2.9) | 42 (2.9) | 520 (35.2) | 956 (64.8) |
| Malawi, 2016 | 1233 | 48 (3.9) | 52 (4.2) | 12 (1.0) | 8 (0.7) | 310 (25.1) | 24 (2.0) | 131 (10.6) | 131 (10.6) | 131 (10.6) | 485 (39.3) | 748 (60.7) |
| Mongolia, 2006 | 242 | 0 (0) | 0 (0) | 0 (0) | 0 (0) | -- | 1 (0.4) | 40 (16.5) | -- | 2 (0.8) | 40 (16.5) | 202 (83.5) |
| Nicaragua, 2005 | 1424 | 11 (0.8) | 10 (0.8) | 2 (0.1) | 2 (0.1) | -- | 4 (0.3) | 4 (0.3) | -- | 0 (0) | 21 (1.5) | 1403 (98.5) |
| Papua New Guinea, 2005 | 934 | 37 (4.0) | 41 (4.4) | 12 (1.3) | 6 (0.6) | -- | 37 (4.0) | 62 (6.6) | 63 (6.7) | 62 (6.6) | 132 (14.1) | 802 (85.9) |
| Zambia, 2009 | 885 | 5 (0.6) | 6 (0.7) | 1 (0.1) | 0 (0) | -- | 2 (0.2) | 494 (55.8) | 473 (53.4) | 474 (53.4) | 572 (64.6) | 313 (35.4) |
| Cameroon, 2009 | 853 | 16 (1.9) | 29 (3.4) | 1 (0.1) | 0 (0) | 195 (22.9) | 3 (0.4) | 61 (7.2) | 61 (7.2) | 61 (.7.2) | 297 (34.8) | 556 (65.2) |
| Nigeria, 2012 | 640 | 61 (9.5) | 63 (9.8) | 30 (4.7) | 25 (3.9) | 198 (30.9) | 13 (2.0) | 63 (9.8) | 93 (14.5) | 93. (14.5) | 357 (55.8) | 283 (44.2) |
| Pakistan, 2011 | 10689 | 1365 (12.8) | 1543 (14.4) | 361 (3.4) | 280 (2.6) | -- | 524 (4.9) | 3370 (31.5) | -- | 3132 (29.3) | 4793 (44.8) | 5896 (55.2) |
| Philippines, 2011 | 1784 | 80 (4.5) | 98 (5.5) | 6 (0.3) | 4 (0.2) | -- | 6 (0.3) | 17 (1.0) | 17 (1.0) | 17 (1.0) | 128 (7.2) | 1656 (92.8) |
| Vietnam, 2010 | 395 | 23 (5.8) | 25 (6.3) | 2 (0.5) | 1 (0.2) | -- | 3 (0.8) | 32 (8.1) | 17 (4.3) | -- | 66 (16.7) | 329 (83.3) |
| Azerbaijan, 2013 | 1404 | 41 (2.9) | 39 (2.8) | 49 (3.5) | 39 (2.8) | -- | 49 (3.5 | 351 (25.0) | 351 (25.0) | 351 (25.0) | 417 (29.7) | 987 (70.3) |
| Colombia, 2010 | 7753 | 66 (0.9) | 76 (1.0) | 11 (0.1) | 7 (0.10) | -- | 161 (2.1) | 3415 (44.0) | 3887 (50.1) | -- | 4042 (52.1) | 3711 (47.9) |
| Mexico, 2012 | 8528 | 101 (1.2) | 102 (1.2) | 0 (0) | 0 (0) | -- | 482 (5.7) | 5932 (69.6) | 5989 (70.2) | -- | 6098 (71.5) | 2430 (28.5) |

^1^Exclusion criteria were: BAZ or WHZ <-2 SD; BAZ or WHZ less than -5 SD or greater than 5 SD;(4) positive malaria test result; or missing values for SR, RBP, CRP, AGP, or BAZ (due to missing values for weight or height/length). Some observations may be excluded from multiple categories. Exclusion criteria percentages are proportions of total observations in the individual datasets. '--’ indicates the variable was not available in that survey. Abbreviations: AGP, alpha-1-acid glycoprotein; BAZ, BMI-for-age z-score; BRINDA, Biomarkers Reflecting Nutritional Determinants of Anemia; CRP, C-reactive protein; RBP, retinol-binding protein; SR, serum retinol; WHZ, weight-for-height z-score.

**Supplemental Table 3.** Available covariates in each survey: BRINDA project^1^

| **Survey** | **Age** | **Sex** | **Rural/Urban Setting** | **SES** | **Water** | **Sanitation** | **Respondent/Maternal**  **Education Level** | **Household Head Education Level** |
| --- | --- | --- | --- | --- | --- | --- | --- | --- |
| ***Women*** |  |  |  |  |  |  |  |  |
| Afghanistan | x | -- | -- | x | x | x | x | -- |
| Azerbaijan | x | -- | x | x | x | x | x | -- |
| Burkina Faso | x | -- | -- | x | x | x | x | x |
| Cambodia | x | -- | x | x | x | x | x | -- |
| Cameroon | x | -- | x | x | x | x | x | -- |
| Côte d’Ivoire | x | -- | x | x | x | x | x | -- |
| Malawi | x | -- | x | x | x | x | x | -- |
| Nigeria | x | -- | x | -- | -- | -- | -- | -- |
| Pakistan | x | -- | x | x | x | x | x | -- |
| Papua New Guinea | x | -- | x | x | -- | -- | x | -- |
| Vietnam | x | -- | x | -- | -- | -- | -- | -- |
| United Kingdom | x | -- | -- | x | -- | -- | x | -- |
| United States | x | -- | -- | x | -- | -- | x | -- |
| ***Children*** |  |  |  |  |  |  |  |  |
| Afghanistan | x | x | -- | x | x | x | x | -- |
| Azerbaijan | x | x | x | x | x | x | x | -- |
| Bangladesh, 2010 | x | x | -- | -- | x | x | -- | -- |
| Bangladesh, 2012 | x | x | x | x | x | x | x | -- |
| Burkina Faso | x | x | -- | x | x | x | x | x |
| Cambodia | x | x | x | x | x | x | x | -- |
| Cameroon | x | x | x | x | x | x | x | -- |
| Colombia | x | x | x | x | x | x | -- | x |
| Côte d’Ivoire | x | x | x | x | x | x | x | -- |
| Kenya, 2007 | x | x | x | x | x | x | x | -- |
| Kenya, 2010 | x | x | x | x | x | x | x | -- |
| Liberia | x | x | x | x | x | x | -- | -- |
| Malawi | x | x | x | x | x | x | x | -- |
| Mexico, 2012 | x | x | x | x | -- | -- | -- | -- |
| Mongolia | x | x | x | -- | -- | -- | x | -- |
| Nicaragua | x | x | x | -- | x | x | x | -- |
| Nigeria | x | x | x | -- | -- | -- | -- | -- |
| Pakistan | x | x | x | x | x | x | x | -- |
| Papua New Guinea | x | x | x | x | x | x | -- | -- |
| Philippines | x | x | x | x | x | x | x | -- |
| Vietnam | x | x | x | -- | -- | -- | -- | -- |
| Zambia | x | x | x | -- | -- | -- | -- | -- |

^1^Covariate definitions: age in years (WRA) or months (PSC); sex (male/female; PSC only); urban or rural residence (surveys from Kenya and Nigeria contained observations from rural areas only); household socioeconomic status (SES, variable created from the ordinal 3-category SES variable from the harmonized BRINDA dataset (5)); water and sanitation sources defined as access to an improved or unimproved water source or toilet facility as defined by the WHO/UNICEF Joint Monitoring Program (6,7); and education level defined as the highest level completed by either the respondent/caregiver or head of household. ‘--’ indicates the variable was unavailable in that survey. Abbreviations: BRINDA: Biomarkers Reflecting Inflammation and Nutritional Determinants of Anemia; SES, socio-economic status.

**Supplemental Table 4.** Age and household characteristics for women (15-49 years) with normal weight to overweight/obesity by survey: BRINDA project^1^

| **CIC^2^** | **Survey, year** | **n** | **Age, yr**  **median [IQR]** | **Urban residence**  **% (95% CI)** | **High SES^3^**  **% (95% CI)** | **Improved Water Source^4^**  **% (95% CI)** | **Improved Toilet^5^**  **% (95% CI)** | **High Education^6^**  **% (95% CI)** |
| --- | --- | --- | --- | --- | --- | --- | --- | --- |
| Low | Afghanistan, 2013 | 571 | 29.2 [24.3, 29.8] | -- | 92.9 (89.2, 96.6) | 81.5 (75.7, 87.4) | 56.6 (47.8, 65.3) | 10.2 (6.1, 14.4) |
|  | Burkina Faso, 2010 | 61 | 30.2 [25.3, 37.7] | -- | 76.5 (57.9, 95.0) | 31.3 (5.8, 56.7) | 13.0 (0.9, 25.1) | 2.1 (0.0, 7.6) |
|  | Cambodia, 2014 | 609 | 29.8 [25.1, 33.6] | 13.1 (9.9, 16.3) | 60.5 (53.7, 67.3) | 53.1 (45.7, 60.6) | 52.1 (46.6, 57.7) | 31.7 (26.5, 36.9) |
|  | Côte d’Ivoire, 2007 | 706 | 26.3 [21.1, 31.9] | 56.1 (51.9, 60.3) | 63.9 (58.1, 69.8) | 88.0 (82.6, 93.3) | 89.9 (85.9, 93.8) | 15.5 (11.5, 19.4) |
|  | Malawi, 2016 | 595 | 27.7 [20.7, 36.7] | 10.3 (1.9, 18.7) | 61.0 (52.9, 69.0) | 83.6 (75.7, 91.4) | 83.8 (76.4, 91.2) | 22.0 (15.6, 28.4) |
|  | Papua New Guinea, 2005 | 692 | 27.6 (20.5, 35.3) | 21.3 (11.1, 31.4) | 61.5 (49.6, 73.5) | -- | -- | 24.4 (19.6, 29.2) |
| Low-middle | Cameroon, 2009 | 594 | 26.2 [22.0, 31.8] | 62.8 (52.5, 73.2) | 64.1 (56.7, 71.6) | 75.0 (68.9, 81.0) | 67.0 (61.5, 72.6) | 37.5 (33.3, 41.7) |
|  | Nigeria, 2012 | 506 | 26.3 [21.5, 31.1] | 0^7^ | -- | -- | -- | -- |
|  | Pakistan, 2011 | 4946 | 29.6 [25.8, 34.6] | 32.7 (29.4, 36.0) | 61.7 (59.1, 64.3) | 92.9 (91.5, 94.2) | 84.5 (82.7, 86.4) | 30.8 (28.5, 33.1) |
|  | Vietnam, 2010 | 1138 | 33.5 [26.0, 41.0] | 49.8 (47.2, 52.5) | -- | -- | -- | -- |
| Upper-middle | Azerbaijan, 2013 | 2528 | 31.7 [23.9, 41.4] | 45.7 (39.4, 51.9) | 68.6 (65.1, 72.2) | 77.1 (72.2, 82.1) | 93.7 (90.9, 96.5) | 95.0 (93.5, 96.5) |
| High | United Kingdom, 2014 | 836 | 33.9 [24.1, 42.0] | -- | 63.7 (58.3, 69.0) | -- | -- | 91.4 (88.7, 94.1) |
|  | United States, 2006 | 2989 | 34.7 [24.7, 42.5] | -- | 74.1 (70.9, 77.3) | - | -- | 100.0 (100,100) |

^1^Estimates account for complex survey design (cluster, strata) with survey weights applied. Inclusion criteria were: BMI ≥18.5 kg/m^2^, not pregnant, and a negative malaria test result to reduce potential confounding from inflammation due to illness or infection. ‘--‘ indicates the variable was unavailable in that survey. Abbreviations: BRINDA, Biomarkers Reflecting Inflammation and Nutritional Determinants of Anemia; CIC, country income classification; SES, socioeconomic status.

^2^CIC defined according to the World Bank definition for the year the survey took place (8).

^3^SES variables was a 3-level ordinal SES variable available from the harmonized BRINDA dataset (5), which was created from survey-specific asset scores (quintiles) of household ownership or composition. ‘High SES’ includes the medium and high levels.

^4^‘Improved Water Source’ was defined as having access to: piped water in a dwelling/yard; a communal/public tap; a borehole/tube well, owned or shared; a protected well/spring; a protected open dug well; or rain water (6).

^5^‘Improved Toilet’ was defined as have access to: a flush toilet/pit latrine flush to piped sewer; a ventilated improved pit/latrine/Sanitation platform; or a flush to pit/latrine (7).

^6^‘High Education’ was defined as completing at least secondary school and was measured as the respondent education level or head of household education level (Burkina Faso).

^7^The survey from Nigeria only contained observations from rural areas.

**Supplemental Table 5.** Age, sex, and household characteristics for children (6-59 months) with normal weight to overweight/obesity by survey: BRINDA project^1^

| **CIC^2^** | **Country, survey year** | **n** | **Age, mo^3^**  **median [IQR]** | **Male**  **%** | **Urban residence**  **% (95% CI)** | **High SES^4^**  **% (95% CI)** | **Improved Water Source^5^**  **% (95% CI)** | **Improved Toilet^6^**  **% (95% CI)** | **High Education^7^**  **% (95% CI)** |
| --- | --- | --- | --- | --- | --- | --- | --- | --- | --- |
| Low | Afghanistan, 2013 | 586 | 27.9 [17.0, 39.9] | 52.2 | -- | 95.1 (92.9, 97.4) | 81.4 (73.8, 88.9) | 68.1 (60.1, 76.2) | 9.9 (5.3, 14.4) |
|  | Bangladesh, 2010 | 1179 | 7.7 [6.3, 9.3] | 49.1 | -- | -- | 98.4 (95.3, 100.0) | 25.2 (16.9, 33.5) | -- |
|  | Bangladesh, 2012 | 360 | 37.9 [26.8, 48.0] | 54.4 | 26.3 (18.5, 34.0) | 44.7 (33.3, 56.0) | 99.0 (97.5, 100.0) | 63.9 (50.0, 77.9) | 47.0 (35.7, 58.2) |
|  | Burkina Faso, 2010 | 60 | 48.3 [43.6, 53.5] | 43.3 | -- | 81.2 (63.9, 98.4) | 31.2 (8.7, 53.6) | 11.3 (0.0, 26.1) | 2.2 (0, 7.4) |
|  | Cambodia, 2014 | 599 | 37.0 [24.6, 48.3] | 54.4 | 11.6 (8.5, 14.7) | 58.4 (50.0, 66.7) | 53.4 (44.4, 62.3) | 53.9 (46.7, 61.1) | 27.7 (22.5, 32.9) |
|  | Côte d’Ivoire, 2007 | 435 | 29.6 [17.3, 44.2] | 54.7 | 58.2 (52.0, 64.5) | 67.7 (62.1, 73.4) | -- | 66.6 (58.0, 75.3) | 12.4 (9.3, 15.4) |
|  | Kenya, 2007 | 665 | 18.6 [12.1, 26.4] | 51.1 | 0^8^ | 60.0 (54.3, 65.8) | 52.6 (42.9, 62.4) | 0.2 (0.0, 0.5) | 14.7 (11.7, 17.7) |
|  | Kenya, 2010 | 551 | 21.7 [13.4, 27.0] | 50.5 | 0^8^ | 61.7 (56.1, 67.2) | 58.2 (49.0, 67.3) | 1.1 (0.1, 2.2) | 16.4 (12.5, 20.3) |
|  | Liberia, 2011 | 956 | 17.3 [11.2, 26.0] | 47.6 | 43.6 (39.0, 48.2) | 68.3 (61.6, 75.0) | 83.2 (61.6, 75.0) | 43.5 (37.0, 50.0) | -- |
|  | Malawi, 2016 | 748 | 31.4 [18.5, 44.7] | 51.3 | 13.7 (0, 28.0) | 56.5 (49.6, 63.3) | 87.2 (81.0, 93.4) | 82.6 (77.5, 87.6) | 24.0 (13.1, 34.9) |
|  | Mongolia, 2006 | 202 | 18.9 [12.3, 26.7] | 53.0 | 46.5 (39.7, 53.4) | -- | -- | -- | 80.3 (74.7, 85.2) |
|  | Nicaragua, 2005 | 1403 | 34.3 [20.2, 46.2] | 50.1 | 56.4 (44.1, 68.7) | -- | 90.2 (85.9, 94.5) | 27.1 (20.4, 33.7) | 37.2 (30.0, 44.4) |
|  | Papua New Guinea, 2005 | 802 | 31.5 [19.6, 44.4] | 53.6 | 19.7 (10.2, 29.1) | 59.2 (47.9, 70.6) | 67.3 (57.7, 76.8) | 8.6 (3.4, 13.7) | -- |
|  | Zambia, 2009 | 313 | 36.6 [24.1, 48.0] | 58.1 | 21.9 (15.8, 28.0) | -- | -- | -- | -- |
| Low-middle | Cameroon, 2009 | 556 | 29.0 [18.8, 38.9] | 50.7 | 61.8 (51.5, 72.1) | 65.8 (57.9, 73.7) | 77.4 (71.4, 83.3) | 67.2 (61.3, 73.1) | 37.7 (32.8, 42.6) |
|  | Nigeria, 2012 | 283 | 29.6 [22.0, 35.8] | 51.6 | 0^7^ | -- | -- | -- | -- |
|  | Pakistan, 2011 | 5896 | 25.5 [14.8, 39.2] | 51.5 | 31.0 (27.9, 34.1) | 58.5 (55.9, 61.1) | 94.7 (93.5, 95.8) | 91.3 (90.2, 92.5) | 20.7 (18.8, 22.6) |
|  | Philippines, 2011 | 1656 | 15.4 [10.7, 19.0] | 49.5 | 9.1 (8.4, 9.8) | 16.0 (12.8, 19.1) | 44.6 (40.0, 49.3) | 92.6 (87.5, 97.7) | 66.6 (61.3, 71.9) |
|  | Vietnam, 2010 | 329 | 37.5 [25.9, 49.2] | 52.0 | 46.2 (40.5, 51.9) | -- | -- | -- | -- |
| Upper-middle | Azerbaijan, 2013 | 987 | 36.5 [23.6, 47.1] | 55.0 | 45.3 (38.1, 52.5) | 68.6 (64.1, 73.1) | 77.4 (71.8, 83.0) | 93.0 (89.9, 96.2) | -- |
|  | Colombia, 2010 | 3711 | 38.3 [26.2, 49.4] | 52.8 | 70.7 (69.4, 72.0) | 48.4 (46.3, 50.5) | 86.3 (83.2, 89.4) | 87.9 (85.1, 90.7) | 42.3 (39.2, 45.4) |
|  | Mexico, 2012 | 2430 | 37.1 [26.3, 49.7] | 49.5 | 72.2 (69.3, 75.2) | 53.5 (49.7, 57.3) | -- | -- | -- |

^1^Estimates account for the complex survey design (cluster, strata) with survey weights applied, except in the survey from Mongolia which followed a simple random sampling design. Inclusion criteria were: BMI-for-age z-score or weight-for-height z-score ≥-2 SD and a negative malaria test result to reduce potential confounding from inflammation due to illness or infection. ‘--‘ indicates the variable was unavailable in that survey. Abbreviations: BRINDA, Biomarkers Reflecting Inflammation and Nutritional Determinants of Anemia; CIC, country income classification; SES, socioeconomic status.

^2^CIC defined according to the World Bank definition for the year the survey took place (8).

^3^Surveys included children aged 6-59 months except the following surveys: Bangladesh (2010), 6-24 months; Kenya (2007, 2010), Liberia, and Mongolia, 6-35 months; Vietnam, 10-59 months; Cameroon and Mexico (2012), 12-59 months; Burkina Faso and the Philippines, 24-59 months.

^4^SES variables was a 3-level ordinal SES variable available from the harmonized BRINDA dataset (5), which was created from survey-specific asset scores (quintiles) of household ownership or composition. ‘High SES’ includes the medium and high levels.

^5^‘Improved Water Source’ was defined as having access to: piped water in a dwelling/yard; a communal/public tap; a borehole/tube well, owned or shared; a protected well/spring; a protected open dug well; or rain water (6).

^6^‘Improved Toilet’ was defined as have access to: a flush toilet/pit latrine flush to piped sewer; a ventilated improved pit/latrine/Sanitation platform; or a flush to pit/latrine (7)

^7^‘High Education’ was defined as completing at least secondary school, and was measured as maternal education level or head of household education level in surveys in which maternal education was not measured (Burkina Faso and Colombia).

^8^The surveys from Kenya (2007 and 2010) and Nigeria only contained observations from rural areas.

**Supplemental Table 6.** Bivariate (B, unadjusted) and multivariable (M, adjusted) percent change associations between Vitamin A, CRP, AGP, and BMI among women (15-49 years) with normal weight to overweight/obesity by survey: BRINDA project^1^

| **CIC** | **Country, survey year** | **RBP or SR** | **n** | **Model** | **Vitamin A**  **regressed on BMI** | **CRP**  **regressed on BMI** | **AGP**  **regressed on BMI** | **Vitamin A**  **regressed on *ln*CRP** | **Vitamin A**  **regressed on *ln*AGP** |
| --- | --- | --- | --- | --- | --- | --- | --- | --- | --- |
| Low-income | Afghanistan, 2013 | SR | 571 | B | 0.2 (-1.4, 1.8) | 5.1 (-2.0, 12.7) | 0.4 (-0.4, 1.2) | 1.4 (-0.4, 3.3) | -1.0 (-8.8, 7.5) |
|  |  |  | 570 | M | 0.2 (-1.4, 1.8) | 4.0 (-3.5, 12.1) | 0.4 (-0.4, 1.2) | 1.4 (-0.4, 3.3) | -1.0 (-8.8, 7.5) |
|  | Burkina Faso, 2010 | SR | 61 | B | -0.1 (-5.5, 5.7) | **-21.8 (-34.3, -7.0)** | -3.5 (-9.9, 3.3) | 0.4 (-9.2, 11.1) | 6.3 (-19.7, 40.7) |
|  |  |  | 61 | M | -0.2 (-5.6, 5.6) | **-22.3 (-35.2, -6.9)** | -4.0 (-9.8, 2.2) | 0.3 (-9.8, 11.5) | 5.4 (-13.3, 53.3) |
|  | Cambodia, 2014 | RBP | 609 | B | -0.9 (-2.3, 0.5) | **17.1 (13.4, 20.9)** | **2.5 (0.5, 4.6)** | **10.5 (6.0, 15.2)** | **62.5 (52.7, 73.0)** |
|  |  |  | 609 | M | -0.7 (-2.1, 0.7) | **16.9 (13.3, 20.6)** | **2.9 (0.8, 5.0)** | **10.4 (5.9, 15.1)** | **62.1 (52.0, 72.9)** |
|  | Côte d’Ivoire, 2007 | RBP | 706 | B | **0.8 (0.3, 1.4)** | **7.3 (4.8, 9.9)** | **2.3 (0.9, 3.8)** | -1.3 (-3.2, 0.7) | -4.7 (-13.1, 4.5) |
|  |  |  | 706 | M | **0.7 (0.1, 1.3)** | **7.1 (4.5, 9.8)** | **2.3 (0.9, 3.8)** | -1.3 (-3.3, 0.7) | -4.7 (-13.0, 4.5) |
|  | Malawi, 2016 | RBP | 595 | B | 1.0 (-0.1, 2.1) | **8.6 (3.1, 14.4)** | **2.9 (1.8, 4.1)** | **-2.7 (-5.0, -0.3)** | -4.6 (-14.8, 6.9) |
|  |  |  | 595 | M | 0.9 (-0.1, 2.0) | **8.6 (3.1, 14.4)** | **2.3 (0.8, 3.7)** | **-3.0 (-5.3, -0.6)** | -3.7 (-14.2, 8.0) |
|  | Papua New Guinea, 2005 | RBP | 692 | B | 0.7 (-0.002, 1.5) | **7.1 (2.8, 11.6)** | 0.4 (-0.2, 1.1) | **-2.6 (-3.9, -1.3)** | -4.5 (-16.0, 8.6) |
|  |  |  | 658 | M | **1.0 (0.2, 1.7)** | **7.1 (2.8, 11.6)** | 0.5 (-0.1, 1.1) | **0.3 (0.04, 0.5)** | -4.4 (-15.9, 8.8) |
| Low-middle | Cameroon, 2009 | RBP | 594 | B | **1.0 (0.4, 1.5)** | **6.5 (3.7, 9.4)** | **0.5 (0.2, 0.9)** | **-1.8 (-3.3, -0.3)** | -2.0 (-16.0, 14.3) |
|  |  |  | 554 | M | **0.8 (0.3, 1.3)** | **6.7 (3.7, 9.8)** | **0.7 (0.3, 1.0)** | **-1.9 (-3.4, -0.4)** | -2.7 (-16.8, 13.8) |
|  | Nigeria, 2012 | RBP | 506 | B | -0.1 (-0.7, 0.6) | -0.6 (-3.5, 2.3) | 0.1 (-0.6, 0.7) | **-4.1 (-6.1, -2.2)** | **14.6 (4.6, 25.6)** |
|  |  |  | 506 | M | -0.1 (-0.7, 0.6) | -0.6 (-3.5, 2.3) | 0.1 (-0.6, 0.7) | **-4.1 (-6.1, -2.2)** | **14.6 (4.6, 25.6)** |
|  | Pakistan, 2011 | SR | 4946 | B | **0.9 (0.3, 1.4)** | **3.0 (1.9, 4.1)** | **1.0 (0.7, 1.3)** | -0.9 (-2.6, 0.9) | 3.6 (-3.7, 11.4) |
|  |  |  | 4851 | M | 0.2 (-0.3, 0.8) | **2.9 (1.8, 4.0)** | **1.0 (0.7, 1.3)** | -1.3 (-3.0, 0.4) | 2.5 (-4.6, 10.1) |
|  | Vietnam, 2010 | SR | 1138 | B | **1.2 (0.3, 2.2)** | **17.4 (14.8, 20.1)** | -- | 1.4 (-0.8, 3.7) | -- |
|  |  |  | 1138 | M | **0.3 (0.02, 0.5)** | **16.6 (13.8, 19.6)** | -- | 1.0 (-1.2, 3.3) | -- |
| Upper-middle | Azerbaijan, 2013 | RBP | 2528 | B | **1.2 (1.0, 1.4)** | **13.6 (12.3, 15.0)** | **1.5 (1.3, 1.8)** | **2.7 (1.9, 3.6)** | **21.6 (15.5, 28.2)** |
|  |  |  | 2506 | M | **1.0 (0.8, 1.2)** | **11.7 (10.2, 13.2)** | **1.5 (1.2, 1.8)** | **1.7 (0.8, 2.6)** | **18.1 (26.2, 39.9)** |
| High | United Kingdom, 2014 | SR | 836 | B | -0.2 (-0.7, 0.3) | **6.5 (5.4, 7.6)** | -- | -1.3 (-5.1, 2.7) | -- |
|  |  |  | 568 | M | -0.3 (-0.8, 0.1) | **6.6 (5.1, 8.0)** | -- | -1.4 (-5.1, 2.5) | -- |
|  | United States, 2006 | SR | 2989 | B | **-0.3 (-0.5, -0.1)** | **11.5 (10.9, 12.1)** | -- | 0.9 (-0.2, 1.9) | -- |
|  |  |  | 2847 | M | **-0.4 (-0.6, -0.1)** | **10.9 (10.3, 11.5)** | -- | 0.6 (-0.5, 1.7) | -- |

^1^Vitamin A (RBP or SR), CRP and AGP variables were *natural-log* (*ln*) transformed for analysis due to non-normal distributions. Regression estimates were exponentiated, and results are presented as the percent change (95% confidence interval) in the dependent variable concentration for every 1-unit change in the independent variable. Note that for the values presented for ‘Vitamin A regressed on *ln*CRP’ and ‘Vitamin A regressed on *ln*AGP’, the percent changes in Vitamin A concentration are for every 1-unit change in *natural-log* transformed CRP or AGP, and the units differ (CRP, mg/L; AGP, g/L). See Table 1 of main manuscript for geometric mean CRP and AGP values by survey. All estimates account for the complex survey design (cluster, strata) with survey weights applied. Vitamin A (as RBP or SR) was measured in either serum or plasma, as reported by the survey. Covariates available for adjustment were: age, education level (respondent or household head), household socioeconomic status, access to an improved water source, access to an improved toilet, and urban/rural residence. Covariates were included in the multivariable regression model if they were associated with the outcome variable at p<0.1 in the bivariate model. Inclusion criteria were: BMI ≥18.5 kg/m^2^, not pregnant (surveys from Cote D’Ivoire, Papua New Guina, Cameroon, Nigeria, and Vietnam did not contain a variable indicating pregnancy, thus results may include data from pregnant women), and a negative malaria test result. CIC was defined according to the World Bank definition for the year in which the survey was conducted (8). ‘—‘ indicates the variable was unavailable in that survey. Abbreviations: AGP, α-1-acid glycoprotein; B, bivariate model; BRINDA, Biomarkers Reflecting Inflammation and Nutritional Determinants of Anemia; CIC; country income classification; CRP, C-reactive protein; *ln*, natural log; M, multivariable model; RBP, retinol binding protein; SR, serum retinol.

**Supplemental Table 7.** Bivariate (B, unadjusted) and multivariable (M, adjusted) percent change associations between Vitamin A, CRP, AGP, and BAZ among children (6-59 months) with normal weight to overweight/obesity by survey: BRINDA project^1^

| **CIC** | **Country, survey year** | **RBP or SR** | **n** | **Model** | **Vitamin A**  **regressed on BAZ** | **CRP**  **regressed on BAZ** | **AGP**  **regressed on BAZ** | **Vitamin A**  **regressed on *ln*CRP** | **Vitamin A**  **regressed on *ln*AGP** |
| --- | --- | --- | --- | --- | --- | --- | --- | --- | --- |
| Low-income | Afghanistan, 2013 | SR | 586 | B | 3.7 (-0.3, 7.8) | -12.3 (-28.1, 7.0) | **-4.0 (-6.2, -1.6)** | **-5.3 (-7.4, -3.1)** | **-31.5 (-38.7, -23.4)** |
|  |  |  | 356 | M | 1.4 (-2.9, 6.0) | -12.3 (-28.1, 7.0) | -3.5 (-7.4, 0.6) | **-5.3 (-8.8, -1.6)** | **-31.2 (-45.3, -13.5)** |
|  | Bangladesh, 2010 | RBP | 1179 | B | **1.4 (0.2, 2.7)** | -3.8 (-11.9, 5.1) | -1.2 (-3.2, 0.9) | **-5.4 (-6.3, -4.5)** | **-22.5 (-27.9, -16.8)** |
|  |  |  | 1165 | M | **1.5 (0.5, 2.6)** | -3.3 (-11.8, 6.1) | -1.0 (-3.0, 1.1) | **-5.3 (-6.0, -5.0)** | **-22.0 (-27.2, -16.5)** |
|  | Bangladesh, 2012 | SR | 360 | B | **4.4 (0.4, 8.5)** | -3.1 (-13.6, 8.8) | -2.2 (-6.3, 2.1) | -4.2 (-11.5, 3.8) | -9.7 (-28.1, 13.4) |
|  |  |  | 303 | M | 3.6 (-0.3, 7.7) | 3.8 (-9.6, 19.2) | -1.4 (-5.2, 2.5) | -2.4(-10.2, 6.0) | -3.4 (-22.5, 20.3) |
|  | Burkina Faso, 2010 | SR | 60 | B | **-9.9 (-18.1, -0.8)** | -6.4 (-42.0, 51.2) | 0.1 (-9.5, 10.6) | **-9.8 (-18.5, -0.1)** | 1.1 (-40.2, 71.0) |
|  |  |  | 60 | M | **-9.9 (-18.4, -0.4)** | -5.1 (-41.1, 53.1) | 0.7 (-7.3, 9.3) | **-10.2 (-19.0, -0.5)** | -2.5 (-43.3, 67.6) |
|  | Cambodia, 2014 | RBP | 599 | B | -2.0 (-8.3, 4.6) | 10.3 (-3.6, 26.3) | 1.7 (-7.0, 11.1) | **3.5 (0.6, 6.5)** | **62.1 (54.4, 70.1)** |
|  |  |  | 599 | M | -2.0 (-8.3, 4.6) | 8.2 (-5.1, 23.2) | 1.7 (-7.0, 11.1) | **3.5 (0.6, 6.5)** | **62.1 (54.4, 70.1)** |
|  | Côte d’Ivoire, 2007 | RBP | 435 | B | -0.3 (-3.0, 2.5) | 8.1 (-7.5, 26.4) | -0.04 (-3.1, 3.2) | **-7.9 (-9.8, -6.0)** | **-25.5 (-32.2, -18.1)** |
|  |  |  | 432 | M | -0.01 (-2.7, 2.8) | 6.3 (-9.1, 24.2) | -1.4 (-4.4, 1.7) | **-7.7 (-9.6, -5.8)** | **-25.2 (-32.2, -17.5)** |
|  | Kenya, 2007 | RBP | 665 | B | 1.0 (-1.7, 3.8) | 4.5 (-7.6, 18.2) | -1.1 (-3.9, 1.7) | **-5.8 (-6.9, -4.7)** | **-22.1 (-26.2, -17.7)** |
|  |  |  | 624 | M | 0.8 (-2.0, 3.8) | 6.9 (-6.1, 21.7) | -1.0 (-3.9, 2.0) | **-5.7 (-6.9, -4.5)** | **-20.6 (-25.3, -15.6)** |
|  | Kenya, 2010 | RBP | 551 | B | -1.8 (-4.6, 1.0) | 7.7 (-9.5, 28.3) | 1.6 (-1.2, 4.5) | **-6.0 (-7.4, -4.7)** | **-26.9 (-32.3, -21.2)** |
|  |  |  | 525 | M | -2.0 (-4.9, 1.0) | 7.7 (-9.5, 28.3) | 1.3 (-1.6, 4.4) | **-6.0 (-7.4, -4.6)** | **-26.7 (-32.6, -20.3)** |
|  | Liberia, 2011 | RBP | 956 | B | 1.1 (-0.9, 3.1) | 3.0 (-7.0, 14.1) | -0.6 (-2.6, 1.4) | **-5.0 (-6.4, -3.6)** | **-21.9 (-29.4, -13.7)** |
|  |  |  | 929 | M | 1.8 (-0.2, 3.8) | 2.0 (-7.8, 12.8) | -0.6 (-2.6, 1.5) | **-5.2 (-6.5, -3.8)** | **-22.5 (-29.4, -14.8)** |
|  | Malawi, 2016 | RBP | 748 | B | 1.4 (-1.3, 4.2) | **18.6 (3.5, 35.8)** | 3.8 (-2.5, 10.6) | **-7.0 (-8.2, -5.7)** | **-12.5 (-18.5, -6.1)** |
|  |  |  | 746 | M | 1.5 (-1.1, 4.2) | **17.2 (2.4, 34.1)** | 3.5 (-2.9, 10.3) | **-6.9 (-8.2, -5.6)** | **-12.4 (-18.3, -6.0)** |
|  | Mongolia, 2006 | SR | 202 | B | -0.9 (-8.9, 7.8) | -- | -2.6 (-6.7, 1.7) | -- | **-24.9 (-42.7, -1.7)** |
|  |  |  | 202 | M | -0.9 (-8.9, 7.8) | -- | -2.9 (-6.9, 1.4) | -- | **-24.9 (-42.7, -1.7)** |
|  | Nicaragua, 2005 | SR | 1403 | B | 0.9 (-1.8, 3.6) | -- | -0.4 (-2.6, 1.8) | -- | **-17.3 (-21.9, -12.4)** |
|  |  |  | 1403 | M | 0.9 (-1.8, 3.6) | -- | -0.4 (-2.5, 1.8) | -- | **-17.3 (-21.9, -12.4)** |
|  | Papua New Guinea, 2005 | RBP | 802 | B | **2.9 (0.5, 5.4)** | -11.7 (-25.3, 4.4) | -2.1 (-4.5, 0.4) | **-6.0 (-7.1, -4.8)** | **-24.7 (-31.3, -17.5)** |
|  |  |  | 369 | M | 2.2 (-0.1, 4.6) | -7.1 (-21.3, 9.6) | -0.8 (-4.2, 2.7) | **-5.8 (-7.0, -4.6)** | **-23.6 (-30.3, -16.3)** |
|  | Zambia, 2009 | SR | 313 | B | -34.5 (-3.5, 3.7) | -13.0 (-37.4, 20.9) | 1.6 (-3.5, 7.0) | **-2.1 (-3.7, -0.4)** | **-15.0 (-23.6, -5.4)** |
|  |  |  | 313 | M | -34.5 (-3.5, 3.7) | -13.0 (-37.4, 20.9) | 1.6 (-3.5, 7.0) | **-2.1 (-3.7, -0.4)** | **-15.0 (-23.6, -5.4)** |
| Low-middle | Cameroon, 2009 | RBP | 556 | B | -1.9 (-4.2, 0.4) | 9.3 (-5.1, 25.9) | -0.4 (-2.3, 1.6) | **-5.7 (-7.0, -4.3)** | **-25.5 (-33.4, -16.6)** |
|  |  |  | 546 | M | -1.9 (-4.2, 0.4) | 12.2 (-2.6, 29.3) | -0.1 (-1.9, 1.7) | **-5.7 (-7.0, -4.3)** | **-25.5 (-33.4, -16.6)** |
|  | Nigeria, 2012 | SR | 283 | B | 1.6 (-1.4, 4.7) | 5.5 (-8.3, 21.4) | -0.2 (-2.8, 2.4) | **-7.0 (-9.1, -4.8)** | **-27.2 (-35.5, -17.7)** |
|  |  |  | 283 | M | 1.6 (-1.4, 4.7) | 5.5 (-8.3, 21.4) | -0.2 (-2.8, 2.4) | **-7.0 (-9.1, -4.8)** | **-27.2 (-35.5, -17.7)** |
|  | Pakistan, 2011 | SR | 5896 | B | -1.5 (-3.4, 0.4) | -- | 0.8 (-0.2, 1.9) | -- | -1.9 (-6.9, 3.4) |
|  |  |  | 5003 | M | -1.6 (-3.6, 0.4) | -- | 1.0 (-0.2, 2.1) | -- | -1.6 (-6.9, 4.1) |
|  | Philippines, 2011 | RBP | 1656 | B | **3.6 (1.6, 5.6)** | **-11.0 (-20.1, -0.9)** | **-2.1 (-3.8, -0.3)** | **-6.9 (-7.7, -6.2)** | **-27.6 (-31.3, -23.8)** |
|  |  |  | 1503 | M | **2.9 (1.0, 4.9)** | -9.2 (-18.7, 1.4) | -1.8 (-3.6, 0.1) | **-6.4 (-7.2, -5.6)** | **-26.0 (-30.1, -21.7)** |
|  | Vietnam, 2010 | SR | 329 | B | 2.6 (-1.3, 6.6) | -8.4 (-20.8, 5.8) | -- | **-4.4 (-7.0, -1.6)** | -- |
|  |  |  | 329 | M | 2.6 (-1.3, 6.6) | -8.4 (-20.8, 5.8) | -- | **-4.4 (-7.0, -1.6)** | -- |
| Upper-middle | Azerbaijan, 2013 | RBP | 987 | B | -0.4 (-2.1, 1.4) | -9.3 (-19.8, 2.5) | -1.9 (-3.9, 0.2) | **-5.8 (-6.8, -4.7)** | **-21.8 (-27.6, -15.6)** |
|  |  |  | 986 | M | -0.2 (-1.9, 1.6) | -8.4 (-18.6, 3.2) | -1.5 (-3.6, 0.5) | **-5.8 (-6.9, -4.7)** | **-22.5 (-28.1, -16.5)** |
|  | Colombia, 2010 | SR | 3711 | B | **2.2 (0.7, 3.8)** | -4.0 (-14.0, 7.1) | -- | **-2.2 (-2.8, -1.6)** | -- |
|  |  |  | 3711 | M | **2.2 (0.7, 3.7)** | -5.5 (-15.2, 5.4) | -- | **-2.1 (-2.7, -1.5)** | -- |
|  | Mexico, 2012 | SR | 2430 | B | 1.2 (-0.4, 3.0) | 7.2 (-3.4, 19.0) | -- | **-6.4 (-7.3, -5.4)** | -- |
|  |  |  | 2430 | M | 1.3 (-0.4, 2.9) | 7.2 (-3.4, 19.0) | -- | **-6.3 (-7.3, -5.4)** | -- |

^1^Vitamin A (RBP or SR), CRP and AGP variables were *natural-log* (*ln*) transformed for analysis due to non-normal distributions. Regression estimates were exponentiated, and results are presented as the percent change (95% confidence interval) in the dependent variable concentration for every 1-unit change in the independent variable. Note that for the values presented for ‘Vitamin A regressed on *ln*CRP’ and ‘Vitamin A regressed on *ln*AGP’, the percent changes in Vitamin A concentration are for every 1-unit change in *natural-log* transformed CRP or AGP, and the units differ (CRP, mg/L; AGP, g/L). See Table 2 of main manuscript for geometric mean CRP and AGP values by survey. All estimates account for the complex survey design (cluster, strata) with survey weights applied. Vitamin A (as RBP or SR) was measured in either serum or plasma, as reported by the survey. Covariates available for adjustment were: age, education level (maternal or household head), household socioeconomic status, access to an improved water source, access to an improved toilet, and urban/rural residence. Covariates were included in the multivariable regression model if they were associated with the outcome variable at p<0.1 in the bivariate model. Inclusion criteria were: BAZ ≥-2 SD, WHZ ≥-2 SD, and a negative malaria test result to reduce potential confounding from inflammation due to illness or infection. CIC was defined according to the World Bank definition for the year in which the survey was conducted (8). ‘--‘ indicates the variable was unavailable in that survey. Abbreviations: AGP, α-1-acid glycoprotein; B, bivariate model; BAZ, BMI-for-age z-score; BRINDA, Biomarkers Reflecting Inflammation and Nutritional Determinants of Anemia; CIC; country income classification; CRP, C-reactive protein; *ln,* natural log; M, multivariable model; RBP, retinol binding protein; SR, serum retinol.

**Supplemental Table 8.** Unadjusted relationships between vitamin A (retinol or RBP) and BMI or BAZ as mediated by inflammation among women (15-49 years) and children (6-59 months) with normal weight to overweight/obesity by survey: BRINDA project^1^

|  |  |  |  | **Women’s mediation analysis, unadjusted^2^** | | | | | | |
| --- | --- | --- | --- | --- | --- | --- | --- | --- | --- | --- |
| **CIC^3^** | **Country, survey year** | **n** | **SR or RBP** | **Total Effect** | | **Direct Effect** | **Indirect Effect** | **% Mediated** | **% Mediated by CRP** | **% Mediated by AGP** |
| Low | Afghanistan, 2013 | 571 | SR | 0.2 (-1.4, 1.8) | | 0.1 (-1.45, 1.8) | 0.1 (-0.1, 0.2) | NM | -- | -- |
|  | Burkina Faso, 2010 | 61 | SR | -0.1 (-4.4, 4.5) | | 0.1 (-5.1, 5.6) | -0.2 (-2.5, 2.2) | NM | -- | -- |
|  | Cambodia, 2014 | 609 | RBP | -0.9 (-2.4, 0.4) | | -2.4 (-3.7, -1.1) | 1.5 (0.2, 2.8) | NM | -- | -- |
|  | Côte d’Ivoire, 2007 | 706 | RBP | 0.8 (0.3, 1.4) | | 1.0 (0.4, 1.6) | -0.2 (-0.30, -0.001) | NM | -- | -- |
|  | Malawi, 2016 | 595 | RBP | 1.0 (-0.1, 2.1) | | 1.3 (0.003, 2.6) | -0.3 (-0.7, 0.1) | NM | -- | -- |
|  | Papua New Guinea, 2005 | 692 | RBP | **0.7 (0.01, 1.5)** | | **0.9 (0.2, 1.6)** | **-0.2 (-0.4, -0.02)** | -26.5 | 82.6 | 17.4 |
| Low-middle | Cameroon, 2009 | 594 | RBP | **1.0 (0.5, 1.5)** | | **1.1 (0.6, 1.6)** | **-0.2 (-0.3, -0.03)** | -15.5 | 81.3 | 18.7 |
|  | Nigeria, 2012 | 506 | RBP | -0.1 (-0.7, 0.5) | | -0.1 (-0.7, 0.5) | 0.1 (-0.2, 0.3) | NM | -- | -- |
|  | Pakistan, 2011 | 4946 | SR | 0.9 (0.3, 1.4) | | 0.9 (0.3, 1.4) | -0.01 (-0.1, 0.1) | NM | -- | -- |
|  | Vietnam, 2010 | 1138 | SR | 1.2 (0.3, 2.2) | | 1.2 (0.1, 2.2) | 0.1 (-0.3, 0.5) | NM | -- | -- |
| Upper-middle | Azerbaijan, 2013 | 2528 | RBP | 1.2 (1.0, 1.4) | | 1.1 (-1.1, 3.4) | 0.1 (-0.1, 0.2) | NM | -- | -- |
| High | United Kingdom, 2014 | 836 | SR | -0.2 (-0.7, 0.3) | | -0.2 (-0.8, 0.4) | -0.1 (-0.4, 0.3) | NM | -- | -- |
|  | United States, 2006 | 2989 | SR | **-0.3 (-0.5, -0.1)** | | **-0.5 (-0.8, -0.3)** | **0.3 (0.1, 0.4)** | -90.7 | 100 | -- |
|  |  |  |  | **Children’s mediation analysis, unadjusted^2^** | | | | | | |
| Low | Afghanistan, 2013 | 306 | SR | 3.7 (-0.2, 7.5) | | 2.2 (-2.0, 6.3) | 1.5 (0.3, 2.6) | NM | -- | -- |
|  | Bangladesh, 2010 | 579 | RBP | 1.4 (0.3, 2.6) | | 1.2 (0.3, 2.1) | 0.3 (-0.3, 0.8 | NM | -- | -- |
|  | Bangladesh, 2012 | 196 | SR | 4.4 (0.5, 8.1) | | 4.3 (0.6, 7.8) | 0.1 (-0.5, 0.8 | NM | -- | -- |
|  | Burkina Faso, 2010 | 26 | SR | -9.9 (-18.3, -2.5) | | -10.7 (-19.7, -3.0) | 1.0 (-4.4, 6.3) | NM | -- | -- |
|  | Cambodia, 2014 | 326 | RBP | -2.0 (-8.5, 4.4) | | -1.5 (-4.6, 1.5) | -0.5 (-5.7, 4.6) | NM | -- | -- |
|  | Côte d’Ivoire, 2007 | 238 | RBP | -0.3 (-3.0, 2.4) | | 0.2 (-1.8, 2.3) | -0.5 (-1.8, 0.7) | NM | -- | -- |
|  | Kenya, 2007 | 340 | RBP | 1.0 (-1.7, 3.7) | | 1.1 (-1.5, 3.6) | -0.04 (-0.8, 0.8) | NM | -- | -- |
|  | Kenya, 2010 | 278 | RBP | -1.9 (-4.6, 0.9) | | -1.3 (-3.9, 1.3) | -0.6 -1.7, 0.5) | NM | -- | -- |
|  | Liberia, 2011 | 455 | RBP | 1.1 (-0.8, 3.1) | | 1.2 (-0.7, 3.0) | -0.1 (-0.7, 0.6) | NM | -- | -- |
|  | Malawi, 2016 | 384 | RBP | 1.4 (-1.2, 4.1) | | 2.7 (0.4, 5.0) | -1.3 (-2.7, 0.2) | NM | -- | -- |
|  | Mongolia, 2006 | 107 | SR | -0.9 (-9.2, 7.5) | | -1.6 (-9.9, 6.6) | 0.8 (-0.7, 2.2) | NM | -- | -- |
|  | Nicaragua, 2005 | 703 | SR | 0.9 (-1.8, 3.5) | | 0.8 (-2.0, 3.5) | 0.1 (-0.3, 0.5) | NM | -- | -- |
|  | Papua New Guinea, 2005 | 430 | RBP | 2.9 (0.6, 5.2) | | 2.2 (-0.03, 4.3) | 0.7 (-0.3, 1.7) | NM | -- | -- |
|  | Zambia, 2009 | 182 | SR | 0.03 (-3.3, 3.4) | | -0.02 (-3.4, 3.4) | 0.1 (-1.1, 1.2) | NM | -- | -- |
| Low-middle | Cameroon, 2009 | 282 | RBP | -1.9 (-4.2, 0.3) | | -1.5 (-3.6, 0.6) | -0.5 (-1.3, 0.4) | NM | -- | -- |
|  | Nigeria, 2012 | 146 | SR | 1.6 (-1.3, 4.4) | 1.9 (-0.8, 4.6) | | -0.3 (-1.3, 0.6) | NM | -- | -- |
|  | Pakistan, 2011 | 3035 | SR | -1.5 (-3.4, 0.4) | -1.5 (-3.4, 0.4) | | -0.01 (-0.1, 0.03) | NM | -- | -- |
|  | Philippines, 2011 | 819 | RBP | **3.6 (1.6, 5.5)** | **-11.0 (-22.3, -1.1)** | | **0.9 (0.1, 1.7)** | 24.3 | 92.5 | 7.4 |
|  | Vietnam, 2010 | 171 | SR | 2.6 (-1.0, 6.1) | 2.2 (-1.3, 5.7) | | 0.4 (-0.2, 1.0) | NM | -- | -- |
| Upper-middle | Azerbaijan, 2013 | 543 | RBP | -0.4 (-2.1, 1.3) | -1.0 (-2.4, 0.4) | | 0.6 (-0.1, 1.3) | NM | -- | -- |
|  | Colombia, 2010 | 1960 | SR | 2.2 (0.7, 3.7) | 2.2 (0.7, 3.6) | | 0.1 (-0.2, 0.3) | NM | -- | -- |
|  | Mexico, 2012 | 1203 | SR | 1.2 (-0.4, 2.9) | 1.7 (0.004, 3.4) | | -0.5 (-1.2, 0.2) | NM | -- | -- |

^1^RBP, SR, CRP, and AGP variables were natural-log (*ln*) transformed for analysis due to non-normal distributions. Mediation estimates were exponentiated, and results are presented as percent change (95% confidence interval) in vitamin A (SR or RBP) concentration for every 1-unit change in BMI (women) or BAZ (children), adjusted for available covariates. SR or RBP concentration measured in serum or plasma, as reported in the survey. All estimates account for cluster survey design (cluster, strata) with survey weights applied, except in the survey from Mongolia which used simple random sampling. Inclusion criteria were: BMI ≥18.5 kg/m2 (women) or BAZ or WHZ ≥-2 SD (children), not pregnant (women only; surveys from Cote D’Ivoire, Papua New Guina, Cameroon, Nigeria, and Vietnam did not contain a variable indicating pregnancy, thus results may include data from pregnant women), and a negative malaria result to reduce potential confounding from inflammation due to illness or infection. Abbreviations: AGP, alpha-1-acid glycoprotein; BAZ, BMI-for-age z-score; BRINDA, Biomarkers Reflecting Inflammation and Nutritional Determinants of Anemia; CIC, country income classification; CRP, C-reactive protein; *ln*, natural log; NM, no mediation; SR, serum retinol; RBP, retinol-binding protein; WHZ, weight-for-height z-score.

^2^Model for mediation analysis: *ln*Vitamin A [RBP or SR] = β_0_ + β_1_(BMI/BAZ) + *M*_1_(*ln*CRP) [+*M*_2_(*ln*AGP)] where all values were continuous and AGP was included as a mediator only in analyses for which it was available in the dataset. Recognizing that models cannot infer causality, the simplified interpretation is as follows: Total Effect = the effect of BMI/BAZ on SR/RBP; Direct Effect = the effect of BMI/BAZ on SR/RBP controlling for inflammation; Indirect Effect = the effect of BMI/BAZ on SR/RBP as mediated by the effect of CRP or AGP. Mediation was considered present when both the total and indirect effects were significant (1,2).

^3^CIC defined according to the World Bank definition for the year in which the survey was conducted (8).

**Supplemental Table 9.** Malaria sensitivity analysis: mediation analysis (unadjusted) assessing the relationship between Vitamin A (RBP or SR), BMI or BAZ and inflammation including and excluding observations that tested positive for malaria for women and children with normal weight to overweight/obesity: BRINDA project^1^

|  | **Including positive malaria observations (*sensitivity analysis*)** | | **Excluding positive malaria observations (*original analysis*)** | |
| --- | --- | --- | --- | --- |
| Survey, year | n | β (95% CI) | n | β (95% CI) |
| *Women* |  | | | |
| Cameroon, 2009 | 691 | -0.002 (-0.003, -0.0006) | 594 | -0.002 (-0.003, -0.0003) |
| Côte d’Ivoire, 2007 | 742 | -0.001 (-0.003, 0.0003) | 706 | -0.002 (-0.003, -0.000008) |
| Malawi, 2016 | 694 | -0.003 (-0.006, -0.0002) | 595 | -0.003 (-0.007, 0.0006) |
| Nigeria, 2012 | 555 | 0.0004 (-0.002, 0.003) | 506 | 0.001 (-0.002, 0.003) |
| *Children* |  | | | |
| Cameroon, 2009 | 740 | -0.002 (-0.01, 0.01) | 556 | -0.01 (-0.01, 0.004) |
| Côte d’Ivoire, 2007 | 606 | -0.004 (-0.02, 0.01) | 435 | -0.005 (-0.02, 0.01) |
| Kenya, 2007 | 825 | -0.003 (-0.01, 0.006) | 665 | -0.0004 (-0.01, 0.01) |
| Kenya, 2010 | 813 | -0.004 (-0.02, 0.01) | 551 | -0.01 (-0.02, 0.01) |
| Liberia, 2011 | 1268 | -0.003 (-0.01, 0.004) | 956 | -0.001 (-0.01, 0.01) |
| Malawi, 2016 | 1027 | -0.02 (-0.03, -0.01) | 748 | -0.01 (-0.03, 0.002) |
| Nigeria, 2012 | 426 | -0.006 (-0.02, 0.03) | 283 | -0.003 (-0.01, 0.01) |
| Zambia, 2009 | 386 | 0.004 (-0.01, 0.02) | 313 | 0.001 (-0.01, 0.01) |

^1^Estimates of the mediated effect are presented as the unexponentiated β coefficient of *ln*Vitamin A (RBP or SR) (95% CI). β represents the indirect effect, that is the effect of BMI (women) or BAZ (children) on Vitamin A (RBP or SR) concentration as mediated by the effect of CRP or AGP. Malaria status was evaluated by survey-specific diagnostic tests that have been previously described (5). While Burkina Faso (2010) measured malaria status, all observations for both women and children were excluded as part of the criteria to exclude observations with underweight/wasting, thus the survey is not included in this sensitivity analysis. Abbreviations: BAZ, BMI-for-age z-score; BRINDA, Biomarkers Reflecting Inflammation and Nutritional Determinant of Anemia; RBP, retinol binding protein; SR, serum retinol.

**Supplemental Table 10.** Morbidity sensitivity analysis: mediation analysis (unadjusted) assessing the relationship between Vitamin A (RBP or SR), BMI or BAZ and inflammation excluding and including observations that reported fever and/or diarrhea in the past 2 weeks (women) and in the past 24 hours (children) with normal weight to overweight/obesity: BRINDA project^1^

|  | **Excluding morbidity observations (*sensitivity analysis*)** | | **Including morbidity observations (*original analysis*)** | |
| --- | --- | --- | --- | --- |
| Survey, year | n | β (95% CI) | n | β (95% CI) |
| *Women* |  | | | |
| Côte d’Ivoire, 2007 | 484 | -0.002 (-0.004, 0.0001) | 706 | -0.002 (-0.003, -0.000008) |
| Malawi, 2016 | 451 | -0.002 (-0.005, 0.002) | 595 | -0.003 (-0.007, 0.0006) |
| *Children* |  | | | |
| Kenya, 2010 | 302 | -0.003 (-0.02, 0.01) | 551 | -0.01 (-0.02, 0.01) |
| Liberia | 221 | 0.002 (-0.01, 0.01) | 956 | -0.001 (-0.01, 0.01) |

^1^Estimates of the mediated effect are presented as the unexponentiated β coefficient of *ln*Vitamin A (RBP or SR) (95% confidence intervals). β represents the indirect effect, that is the effect of BMI (women) or BAZ (children) on Vitamin A (RBP or SR) concentration as mediated by the effect of CRP or AGP. Morbidity status was self-reported by the participants (women) and reported by caregivers of children or measured directly (i.e., fever) as indicated by the survey (5). Inclusion criteria were: BMI ≥18.5 kg/m2 (women) or BAZ or WHZ ≥-2 SD (children), not pregnant (women only; survey from Cote D’Ivoire did not contain a variable indicating pregnancy, thus results may include data from pregnant women), and a negative malaria result to reduce potential confounding from inflammation due to illness or infection. Abbreviations: BAZ, BMI-for-age z-score; BRINDA, Biomarkers Reflecting Inflammation and Nutritional Determinant of Anemia; RBP, retinol binding protein; SR, serum retinol.

**Supplemental Table 11.** Sensitivity mediation analysis examining relationships among both biomarkers of vitamin A (retinol binding protein or serum retinol) and BMI as mediated by inflammation among women (15-49 years) and children (6-59 months) with normal weight to overweight/obesity by survey: BRINDA project^1^

| **Country, survey year** | **n** | **RBP or SR** | **Total Effect** | **Direct Effect** | **Indirect Effect** | **% Mediated** | **% Mediated by CRP** | **% Mediated by AGP** |
| --- | --- | --- | --- | --- | --- | --- | --- | --- |
|  |  |  | **Women’s mediation analysis, adjusted** | | | | | |
| Burkina Faso, 2010 | 61 | RBP* | -5.9 (-12.9, 1.6) | -4.1 (-10.1, 2.2) | -1.9 (-4.8, 1.2) | NM | -- | -- |
|  | 61 | SR | -0.2 (-4.5, 4.4) | -0.03 (-5.5, 5.7) | -0.1 (-2.7, 2.4) | NM | -- | -- |
| Cameroon, 2009 | 594 | RBP | **0.8 (0.3, 1.4)** | **1.0 (0.4, 1.5)** | **-0.2 (-0.3, -0.03)** | -17.8% | 82.6% | 17.4% |
|  | 82 | SR* | 1.0 (-0.4, 2.5) | 1.2 (-0.2, 2.5) | -0.1 (-0.7, 0.5) | NM | -- | -- |
| Malawi, 2016 | 595 | RBP | 0.9 (-0.1, 1.9) | 1.2 (-0.04, 2.4) | -0.3 (-0.5, 1.3) | NM | -- | -- |
|  | 65 | SR* | **3.8 (1.4, 6.2)** | 0.0 (-0.2, 0.2) | **3.8 (1.4, 6.3)**** | 99.9% | 100% | 0% |
| Nigeria, 2012 | 506 | RBP | -0.1 (-0.7, 0.5) | -0.1 (-0.7, 0.5) | 0.1 (-0.2, 0.3) | NM | -- | -- |
|  | 502 | SR* | 1.0 (0.2, 1.9) | 1.0 (0.7, 1.9) | 0.0 (-0.1, 0.1) | NM | -- | -- |
|  |  |  | **Children’s mediation analysis, adjusted** | | | | | |
| Burkina Faso, 2010 | 63 | RBP* | -5.4 (-14.4, 3.3) | -6.6 (-12.6, -1.1) | 1.7 (-6.5, 9.2) | NM | -- | -- |
|  | 60 | SR | -9.9 (-18.5, -2.3) | -10.7 (-19.7, -3.0) | 1.0 (-4.3, 6.2) | NM | -- | -- |
| Cameroon, 2009 | 556 | RBP | -1.9 (-4.2, 0.3) | -1.5 (-3.6, 0.6) | -0.5 (-1.3, 0.4) | NM | -- | -- |
|  | 77 | SR* | -4.7 (-13.3, 3.6) | -5.4 (-14.3, 3.2) | 0.7 (-2.4, 3.9) | NM | -- | -- |
| Malawi, 2016 | 748 | RBP | 1.5 (-1.1, 4.1) | 2.8 (0.4, 5.0) | -1.3 (-2.3, -0.3) | NM | -- | -- |
|  | 54 | SR* | 13.6 (3.1, 22.4) | 10.7 (-0.2, 20.5) | 2.7 (-3.3, 8.6) | NM | -- | -- |
| Nigeria, 2012 | 303 | RBP* | 2.6 (-0.3, 5.4) | 3.3 (0.5, 6.0) | -0.7 (-1.7, 0.3) | NM | -- | -- |
|  | 283 | SR | 1.6 (-1.3, 4.4) | 1.9 (-0.8, 4.6) | -0.3 (-1.3, 0.6) | NM | -- | -- |

*Indicates the vitamin A biomarker included in the sensitivity analysis.

**P-value for indirect effect was 0.01.

^1^ The vitamin A biomarkers selected for the original analysis was the biomarker with more observations prior to application of our exclusion criteria, meaning that the sensitivity analysis vitamin A biomarker may have a larger n than the original analysis. All models were adjusted for covariates. Covariates available for adjustment were: age, education level (head of household or maternal education level), household socioeconomic status, access to an improved water source, access to an improved toilet, and urban/rural residence. Covariates were included in the mediation model if they were associated with the outcome variable at p<0.1 in bivariate models (Supplemental Table 7). SR, RBP, and inflammation biomarkers CRP and AGP variables were natural-log transformed for analysis due to non-normal distributions. Mediation estimates were exponentiated, and results are presented as percent change (95% confidence interval) in vitamin A (SR or RBP) concentration for every 1-unit change in BMI or BAZ, adjusted for available covariates. SR or RBP concentration measured in serum or plasma, as reported in the survey. All estimates account for cluster survey design (cluster, strata) with survey weights applied. Inclusion criteria were: women: BMI ≥18.5 kg/m^2^, not pregnant, and a negative malaria test result; children: BAZ or WHZ ≥-2 SD and a negative malaria test result to reduce potential confounding from inflammation due to illness or infection. Model for mediation analysis: *ln*Vitamin A [RBP or SR] = β_0_ + β_1_(BMI or BAZ) + *M*_1_(*ln*CRP) [+*M*_2_(*ln*AGP)] where all values were continuous and CRP or AGP were included as a mediator only in analyses for which it was available in the dataset. Recognizing that models cannot infer causality, the simplified interpretation is as follows: Total Effect = the effect of BMI or BAZ on vitamin A (retinol or RBP) not controlling for inflammation; Direct Effect = the effect of BMI or BAZ on vitamin A (retinol or RBP) controlling for inflammation; Indirect Effect = the effect of BMI or BAZ on vitamin A (retinol or RBP) mediated by the effect of CRP and/or AGP. Mediation was considered present when both the total and indirect effects were significant (1, 2). Abbreviations: AGP, alpha-1-acid glycoprotein; BAZ, BMI-for-age z-score; BRINDA, Biomarkers Reflecting Inflammation and Nutritional Determinants of Anemia; CRP, C-reactive protein; NM, no mediation WHZ, weight-for-height z-score.

**SUPPLEMENTAL REFERENCES**

1. Baron RM, Kenny DA. The moderator-mediator variable distinction in social psychological research: conceptual, strategic, and statistical considerations. J Pers Soc Psychol. 1986;51(6):1173–82.

2. Hayes AF. Beyond Baron and Kenny: Statistical Mediation Analysis in the New Millennium. Communication Monographs. 2009 Dec 1;76(4):408–20.

3. Williams AM, Guo J, Addo OY, Ismaily S, Namaste SML, Oaks BM, et al. Intraindividual double burden of overweight or obesity and micronutrient deficiencies or anemia among women of reproductive age in 17 population-based surveys. Am J Clin Nutr. 2020 Aug 1;112(Supplement_1):468S-477S.

4. Engle-Stone R, Guo J, Ismaily S, Addo OY, Ahmed T, Oaks B, et al. Intraindividual double burden of overweight and micronutrient deficiencies or anemia among preschool children. Am J Clin Nutr. 2020 Aug 1;112(Supplement_1):478S-487S.

5. Namaste SM, Aaron GJ, Varadhan R, Peerson JM, Suchdev PS. Methodologic approach for the Biomarkers Reflecting Inflammation and Nutritional Determinants of Anemia (BRINDA) project. Am J Clin Nutr. 2017 Jul 1;106(suppl_1):333S-347S.

6. WHO/UNICEF. Drinking water, WHO/UNICEF Joint Monitoring Programme [Internet]. [cited 2022 Jun 14]. Available from: https://washdata.org/monitoring/drinking-water

7. WHO/UNICEF. Sanitation, WHO/UNICEF Joint Monitoring Programme [Internet]. [cited 2022 Jun 14]. Available from: https://washdata.org/monitoring/sanitation

8. The World Bank. World Development Indicators - The World by Income and Region [Internet]. [cited 2021 Apr 29]. Available from: https://datatopics.worldbank.org/world-development-indicators/the-world-by-income-and-region.html
